# The *Pseudomonas aeruginosa* Type III Translocon Is Required for Biofilm Formation at the Epithelial Barrier

**DOI:** 10.1371/journal.ppat.1004479

**Published:** 2014-11-06

**Authors:** Cindy S. Tran, Stephanie M. Rangel, Henrik Almblad, Arlinet Kierbel, Michael Givskov, Tim Tolker-Nielsen, Alan R. Hauser, Joanne N. Engel

**Affiliations:** 1 Department of Pediatrics, University of California San Francisco, San Francisco, California, United States of America; 2 Department of Microbiology-Immunology, Northwestern University, Chicago, Illinois, United States of America; 3 Costern Biofilm Center, Department of International Health, Immunology and Microbiology, Faculty of Health Sciences, University of Copenhagen, Copenhagen, Denmark; 4 Institut Pasteur de Montevideo, Montevideo, Uruguay; 5 Singapore Centre on Environmental Life Sciences Engineering, Nanyang Technological University, Singapore; 6 Department of Microbiology and Immunology, University of California San Francisco, San Francisco, California, United States of America; 7 Department of Medicine, University of California San Francisco, San Francisco, California, United States of America; University of Iowa, United States of America

## Abstract

Clinical infections by *Pseudomonas aeruginosa*, a deadly Gram-negative, opportunistic pathogen of immunocompromised hosts, often involve the formation of antibiotic-resistant biofilms. Although biofilm formation has been extensively studied *in vitro* on glass or plastic surfaces, much less is known about biofilm formation at the epithelial barrier. We have previously shown that when added to the apical surface of polarized epithelial cells, *P. aeruginosa* rapidly forms cell-associated aggregates within 60 minutes of infection. By confocal microscopy we now show that cell-associated aggregates exhibit key characteristics of biofilms, including the presence of extracellular matrix and increased resistance to antibiotics compared to planktonic bacteria. Using isogenic mutants in the type III secretion system, we found that the translocon, but not the effectors themselves, were required for cell-associated aggregation on the surface of polarized epithelial cells and at early time points in a murine model of acute pneumonia. In contrast, the translocon was not required for aggregation on abiotic surfaces, suggesting a novel function for the type III secretion system during cell-associated aggregation. Supernatants from epithelial cells infected with wild-type bacteria or from cells treated with the pore-forming toxin streptolysin O could rescue aggregate formation in a type III secretion mutant, indicating that cell-associated aggregation requires one or more host cell factors. Our results suggest a previously unappreciated function for the type III translocon in the formation of *P. aeruginosa* biofilms at the epithelial barrier and demonstrate that biofilms may form at early time points of infection.

## Author Summary

Clinical infections by *Pseudomonas aeruginosa*, a deadly Gram-negative, opportunistic pathogen of immunocompromised patients, involve the formation of antibiotic-resistant biofilms. Although *P. aeruginosa* biofilm formation has been extensively studied on glass or plastic surfaces, less is known about biofilm formation at the epithelial barrier. This study shows that, on epithelial cells, *P. aeruginosa* forms aggregates that exhibit key characteristics of biofilms. Furthermore, we demonstrate that aggregation on epithelial cells and at early time points in mouse pneumonia requires pore formation mediated by the type III secretion system. Our results indicate that biofilm-like aggregation is induced by a host cell factor that is released after pore formation, suggesting an unexpected role for an acute virulence factor in biofilm formation.

## Introduction

For *Pseudomonas aeruginosa* and many other human pathogens, biofilms are an important mode of growth in a wide variety of clinically significant infections, including lung infections in cystic fibrosis patients, endocarditis, and periodontitis [1], [2]. A widely accepted and core definition of a biofilm comprises: 1) the presence of aggregates or microcolonies that adhere to a surface, 2) encasement in an extracellular matrix, and 3) increased resistance to antimicrobials [1]–[3]. This last feature has profound clinical implications and is thought to account for many infectious disease challenges, including recurrent or persistent infections and the spread of infectious emboli [4]. Thus, models of human pathogenic infections that incorporate biofilms and host cells are essential for developing rational approaches to prevention and treatment.

The Gram-negative, opportunistic pathogen *P. aeruginosa* causes both life-threatening hospital-acquired acute infections—including ventilator-associated pneumonia, post-operative wound infections, and skin and soft tissue infections in burn patients—as well as chronic lung infections in patients with cystic fibrosis. Previous reports support the concept that acute infections are associated with the planktonic life style, whereas chronic infections involve biofilm formation in the host [5]. For example, critical virulence factors associated with acute infections, such as the type III secretion system (T3SS)—a contact-dependent, needle-like apparatus that directly injects bacterial toxins into infected host cells—are inversely regulated with biofilm determinants, such as the production of the exopolysaccharides Psl and Pel [6]. Some recently published data challenges this idea [7]–[9].

However, most of what is known about *P. aeruginosa* biofilm formation and development derives from studies on abiotic (i.e. glass or plastic) surfaces. Although relevant to device-related infections, whether these findings are pertinent to biofilms formed at the epithelial barrier, such as in lung tissue, are important unanswered questions. Only a few groups have investigated *P. aeruginosa* biofilm formation on epithelial cells [10], [11]. While that work has shown that host cells play an important role in the development of biofilms, the bacterial virulence determinants of *P. aeruginosa* biofilm formation on eukaryotic cells have not been fully examined.

We have previously used *P. aeruginosa* infection of polarized epithelial cells to model host-pathogen interactions at the mucosal barrier [12], [13]. When grown for several days on porous membrane filters (Transwells), Madin-Darby Canine Kidney (MDCK) epithelial cells form well-polarized, confluent monolayers with distinct apical and basolateral surfaces that are separated by tight junctions [14]. When *P. aeruginosa* is added to the apical surface of polarized epithelial cells, cell-associated aggregates are formed from free-swimming planktonic bacteria within 30 minutes [15], [16]. The binding of cell-associated aggregates induces remarkable changes in epithelial cell polarity that result in the formation of an actin-rich host cell protrusion at the site of aggregate binding [15], [17]. This model system allows clinically important questions to be asked about the mechanism and requirements of cell-associated aggregation during infection at the epithelial barrier, which can then be extended to *in vivo* models of human disease.

We used this epithelial cell model system together with a murine model of acute *P. aeruginosa* pneumonia to investigate whether cell-associated aggregates of *P. aeruginosa* show characteristics of biofilms and whether biofilm formation is modulated by the T3SS. Our studies reveal a previously unappreciated role of the T3SS in the formation of biofilms at the epithelial barrier. Furthermore, our results indicate that biofilm formation may occur at early time points of infection, thus opening up new therapeutic strategies for treating acute *P. aeruginosa* infections.

## Results

### Cell-associated aggregates represent biofilms

**Figure 1:**
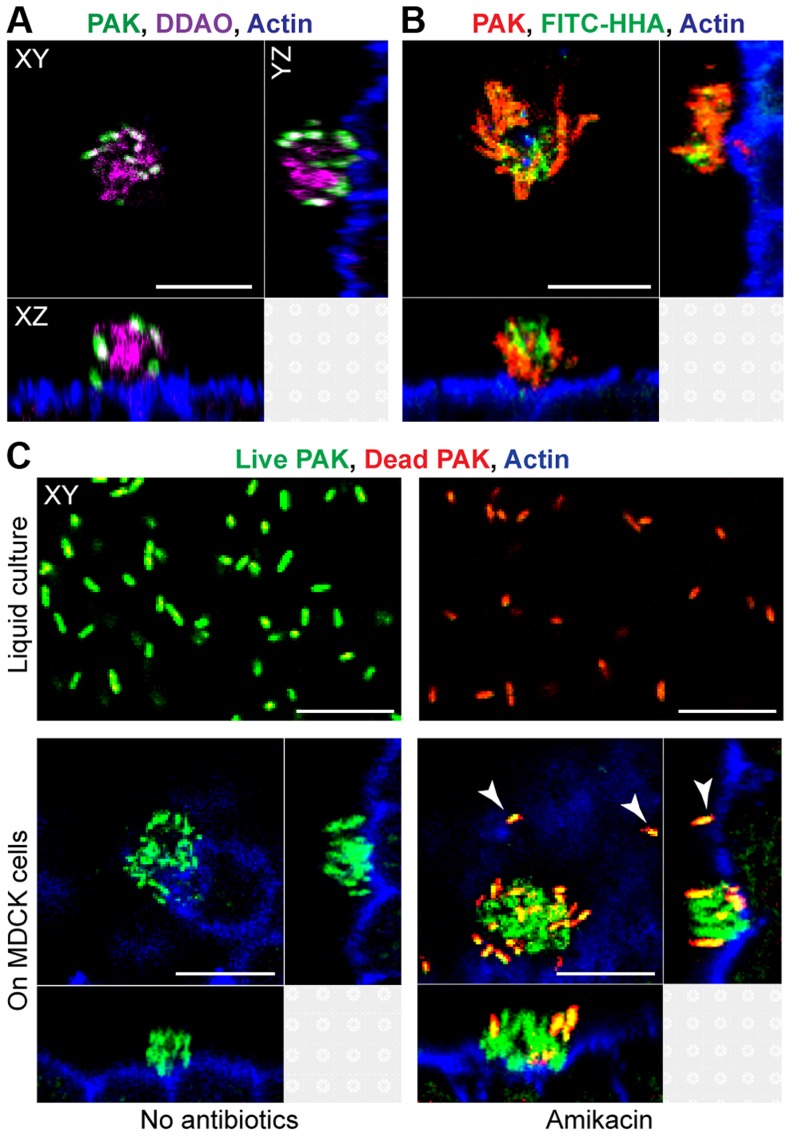
Cell-associated aggregates show key characteristics of biofilms. (A) MDCK cells were infected with PAK-GFP (green), fixed, and stained for actin (blue) and extracellular DNA with DDAO (purple). (B) MDCK cells were infected with PAK-mCherry (red), fixed, and stained for actin (blue) and with FITC-HHA, which binds to Psl. (C) Bacterial viability after exposure to amikacin was evaluated by staining live bacteria with SYTO 9 (green) and counterstaining dead bacteria with propidium iodide (red). MDCK cells were also stained for actin (blue). Bacteria were incubated in liquid culture (MEM, top row) or with MDCK cells (bottom row). After 60 minutes of infection, bacteria in liquid culture or on MDCK cells were treated with amikacin (400 ug/ml) for 2 hours (top right and bottom right panels). Adherent single bacteria showed propidium iodide staining after amikacin treatment (white arrows). Representative confocal images from three independent experiments are shown. Scale bars, 10 µm.

We first tested whether cell-associated aggregates showed key characteristics of biofilms, including the presence of a matrix containing extracellular DNA (eDNA) and exopolysaccharides, increased resistance to antimicrobials, and a requirement for the major adhesins type IV pili and flagella [3], [18]. Confluent monolayers of MDCK epithelial cells were apically infected with the *P. aeruginosa* strain PAK or PAO1, fixed after 60 minutes of infection, and examined by laser scanning confocal microscopy together with fluorescent probes for DNA and polysaccharides. Incubation with DDAO [19], a nucleic acid stain, revealed a cloud of eDNA within and surrounding the aggregate (Figure 1A). The aggregates also stained with the lectins FITC-HHA (Figures 1B and S1B), which binds to the exopolysaccharide Psl [20], [21], and FITC-Concanavalin A (Figures S1A and B), which binds to mannose-containing polysaccharides such as alginate. The observed lectin staining of bacterial aggregates was specific, as no FITC-HHA or FITC-Concanavalin A staining was observed in PAO1Δ*pelFpslD*, a strain lacking two major *P. aeruginosa* exopolysaccharides (Figure S1B).

To determine whether cell-associated aggregates showed increased resistance to antibiotics, PAK was incubated with high doses of amikacin (400 ug/ml, >50 times the MIC_50_) for 2 hours, fixed, and subjected to live (green)/dead (red) staining. Although 100% of planktonically grown bacteria were killed after 2 hour exposure to amikacin, only a small fraction of the bacteria in cell-associated aggregates were killed by amikacin (red/orange staining), while most remained viable (Figure 1C). Single bacteria bound to MDCK cells were uniformly killed by amikacin (white arrows). These findings indicate that cell-associated wild-type aggregates exhibit resistance to high doses of amikacin and suggest that antibiotic resistance is specific to cell-associated aggregates, but not to adherent single bacteria. Live/dead staining of PA01Δ*pelFpslD* aggregates after 2 hour exposure to amikacin showed that all bacteria were readily killed (Figure S1C), further suggesting that antibiotic resistance of cell-associated aggregates requires exopolysaccharide production.

We next sought to determine the role of flagella and type IV pili in cell-associated aggregation, as these two *P. aeruginosa* adhesins are required for abiotic biofilm formation [18], [22]. To quantify cell-associated aggregation, we recorded the number of cell-associated aggregates (defined as groupings of ≥10 bacteria) formed after 60 minutes of infection by imaging 342 consecutive fields (5*10^6^ square microns) in the center of each monolayer with spinning disk confocal microscopy. Formation of cell-associated aggregates was reduced by 90–95% in both the non-flagellated mutant PAKΔ*fliC* and the non-piliated mutant PAKΔ*pilA* (Figure S2). Together, these results support the hypothesis that cell-associated aggregates represent *P. aeruginosa* biofilms.

### Cell-associated aggregation requires the T3SS translocon but does not require T3SS effectors

The T3SS is a key virulence determinant in acute infections *in vitro* and *in vivo* [23], but most published studies suggest that it is downregulated in abiotic biofilms [6], [9], [24], [25]. To investigate the role of T3SS components in cell-associated aggregates, we infected MDCK cells stably expressing the PH domain of Akt fused to GFP (PH-Akt-GFP), a probe for the basolateral phospholipid phosphatidylinositol 3,4,5-trisphosphate (PIP3) [26], with a panel of isogenic T3SS mutants. This cell line allowed us to visualize host cell protrusions at the site of aggregate binding [15], [17]. The bacterial strains tested were PAKΔ*STY*, which lacks all known secreted effectors of PAK; PAKΔ*pscC*, which harbors a mutation in a structural gene for the T3SS needle apparatus and therefore cannot form a needle complex or a translocon; and PAKΔ*popB*, which harbors a mutation in an essential component of the pore-forming translocon but has a functional T3SS needle apparatus [27].

**Figure 2:**
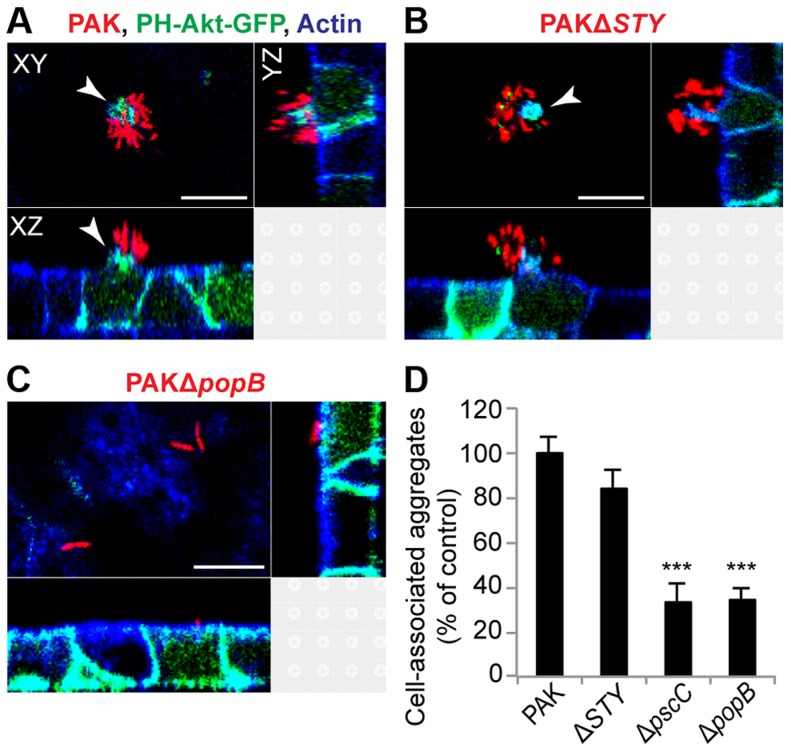
Cell-associated aggregation requires the T3SS translocon but does not require T3SS effectors. MDCK cells stably transfected with PH-Akt-GFP (green) were infected with mCherry-expressing (A) PAK, (B) PAKΔ*STY* (lacks the known T3SS effectors), or (C) PAKΔ*popB* (has the functional needle apparatus but lacks the translocon) for 60 minutes, fixed, and stained for actin (blue). Cell-associated aggregates (red) were visible with PAK (A) and PAKΔ*STY* (B) and were accompanied by formation of membranous protrusions (white arrows) containing PH-Akt-GFP. PAK*ΔpopB* (C) bound individually or as groups of 2 to 3 bacteria. Representative confocal images from three independent experiments are shown. Scale bars, 10 µm. (D) Cell-associated aggregation by PAK and T3SS mutants was quantified using spinning disk confocal microscopy. Shown is the number of aggregates (≥10 bacteria) normalized to PAK (n≥3 independent experiments). Data are mean ± SEM. ***p<0.001 compared to PAK. Statistics in Supplemental Statistical Analysis (Text S1).

We first observed that the effectorless mutant PAKΔ*STY* could form cell-associated aggregates similar to those formed by PAK (Figures 2A and 2B). Furthermore, these aggregates were associated with the formation of membranous protrusions on the apical surface of MDCK cells that were enriched in PIP3, as occurs with wild-type bacteria [17]. Quantification by spinning disk confocal microscopy demonstrated that the efficiency of cell-associated aggregation was similar in PAK and PAKΔ*STY* (Figure 2D). Thus, the three known T3SS effectors encoded in PAK are not required for cell-associated aggregate or protrusion formation. In contrast, PAKΔ*pscC* and PAKΔ*popB* showed a 66% decrease in cell-associated aggregation (Figure 2D). Importantly, overall binding was not decreased: individual or groups of 2 to 3 bacteria were observed to bind to the cell surface (Figure 2C, Figure S3A), and the number of bound colony-forming units (CFUs) per well was similar in all mutants when measured by conventional adhesion assays (Figure S3B), consistent with previous reports [28]. The few aggregates that were formed by PAKΔ*popB* resembled wild-type aggregates in production of eDNA (Figure S3C) and exopolysaccharide (Figure S3D and E) and in antibiotic resistance (Figure S3F). Overall, our results show that the T3SS translocon is required for cell-associated aggregation, whereas the known T3SS effectors are not required.

### Bacterial aggregation in mouse pneumonia requires the T3SS translocon but does not require T3SS effectors

**Figure 3:**
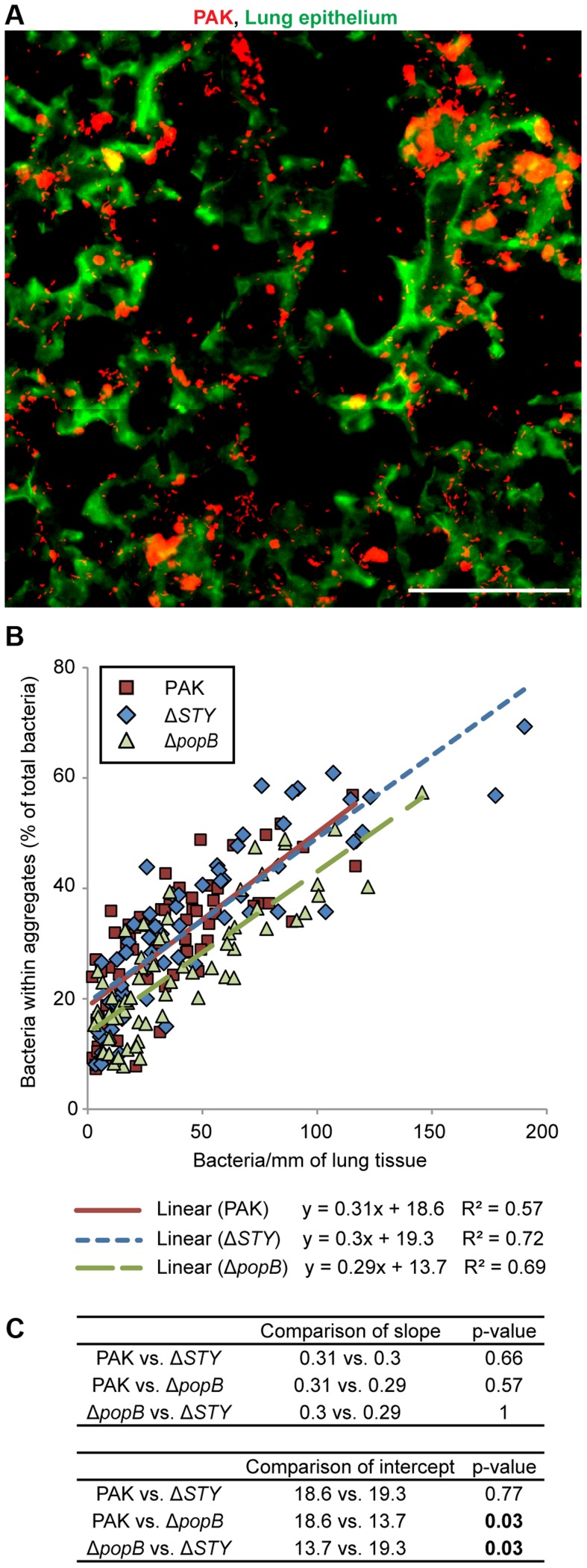
Bacterial aggregation in mouse pneumonia requires the T3SS translocon but does not require T3SS effectors. BALB/c mice were injected intranasally with PAK, PAKΔ*STY*, or PAKΔ*popB* and lungs were isolated, sectioned, and stained at 3 hours post-infection. (A) Widefield epifluorescent imaging showed PAK (red) bound to lung epithelium (green). Representative image from 67 images is shown. Scale bar, 100 µm. (B) The amount of aggregation by PAK and T3SS mutants was quantified and plotted against bacterial density (n≥60 images for each strain). Linear regression lines were applied to each bacterial strain. (C) Comparisons of linear regression lines showed no differences in slope but significant differences in the intercept of PAKΔ*popB* compared to PAK or PAKΔ*STY*. Statistics in Supplemental Statistical Analysis (Text S1).

To determine whether the T3SS also plays a role in bacterial aggregation *in vivo*, mice were intranasally infected with PAK and the T3SS mutants PAKΔ*STY* and PAKΔ*popB*. Lungs were isolated, sectioned, and stained with anti-Pseudomonal antibody at 3 hours post-infection to examine differences in aggregation while avoiding cytotoxicity to airway cells and the subsequent inflammatory response expected at later time points [29]. Overall bacterial load (measured as CFUs/lung) was similar for all strains (Figure S4A and B). Imaging of lung sections by widefield epifluorescent microscopy (Figure 3A) and by confocal microscopy (Figure S4C) after infection with PAK revealed the formation of multiple bacterial aggregates attached to the epithelium of distal airways and alveoli. Microscopic images of PAKΔ*STY* also showed robust aggregation, whereas less aggregation was observed with PAKΔ*popB* (Figure S4D).

Because intranasal infection results in “pockets” of high and low bacterial density in the distal airways, over 60 widefield images of varying bacterial density were analyzed for each bacterial strain. By quantifying the proportion of total bacteria that was found within aggregates, we discovered that the amount of aggregation increased linearly with bacterial density across all strains. (Figure 3B and Figure S4E). However, compared to PAK and PAKΔ*STY*, PAKΔ*popB* showed a ∼30% decrease in aggregation as judged by pairwise comparisons using linear regression lines (Figure 3C, p<0.05). Thus, our results demonstrate that the early stages of aggregation in a murine model of pneumonia are dependent on the T3SS translocon but independent of the T3SS effectors. These findings suggest that our model system of aggregate formation on MDCK cells can be used to accurately model *in vivo* events.

### Co-infection with type III secretion-competent bacteria restores cell-associated aggregation in the translocon mutant

**Figure 4:**
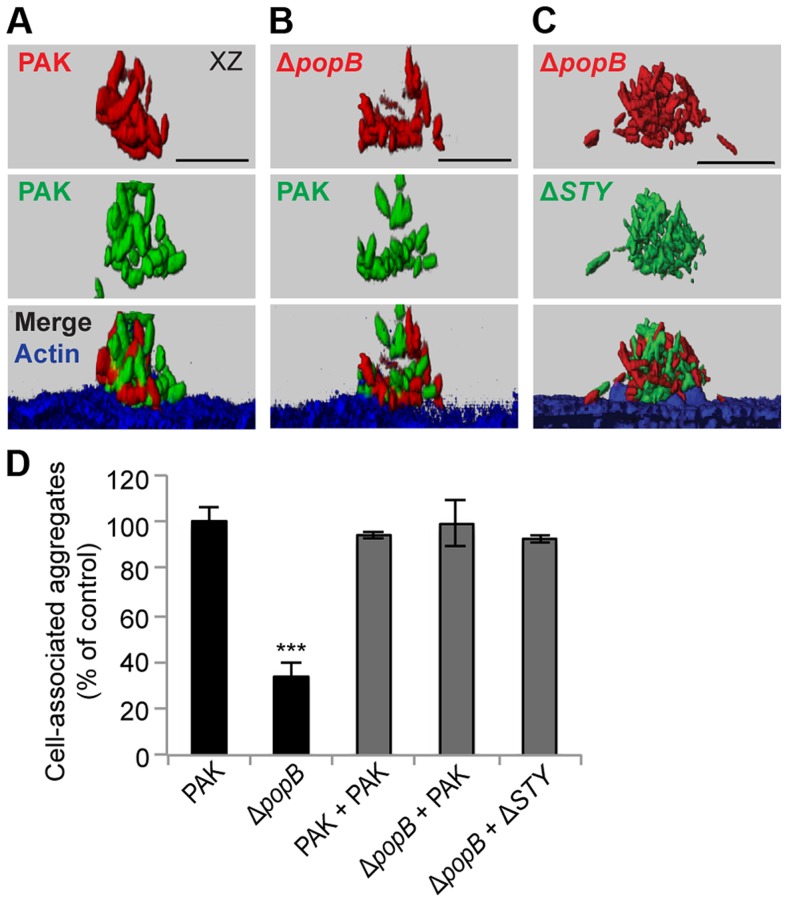
Co-infection with T3SS+ bacteria restores cell-associated aggregation in PAKΔ*popB*. MDCK cells were co-infected with equal amounts of mCherry-expressing (red) and GFP-expressing bacteria (green), fixed, and stained for actin (blue). Co-infection with (A) PAK-mCherry and PAK-GFP, (B) PAKΔ*popB*-mCherry and PAK-GFP, and (C) PAKΔ*popB*-mCherry and PAKΔ*STY*-GFP resulted in cell-associated aggregates composed equally of both strains of bacteria. Representative 3-D reconstructions from three independent experiments are shown. Scale bars, 10 µm. (D) Cell-associated aggregation after co-infection was quantified using spinning disk confocal microscopy. Shown is the number of aggregates (≥10 bacteria) normalized to PAK (n≥3 independent experiments). Data are mean ± SEM. ***p<0.001 compared to PAK. Statistics in Supplemental Statistical Analysis (Text S1).

To elucidate the mechanism responsible for differences in aggregation among T3SS mutants, we considered the possibility that type III translocon-mediated membrane damage could result in the release of a factor that could act in *trans* to induce bacterial aggregation. We therefore assayed by confocal microscopy whether the defect in cell-associated aggregation of PAKΔ*popB* could be rescued by co-infection with T3SS+ bacteria. Equal numbers of PAKΔ*popB* expressing mCherry and T3SS+ bacteria expressing GFP were added to the apical surface of MDCK cells and the composition of the resulting aggregates was determined by volumetric analysis. In control experiments, mixing of PAK-mCherry with PAK-GFP led to the formation of cell-associated aggregates composed equally of red and green bacteria (Figure 4A), consistent with previous reports that aggregates form *de novo* after contact with the cell surface [16]. When PAKΔ*popB*-mCherry was co-infected with PAK-GFP, the deficiency in cell-associated aggregation was restored (Figure 4D), and the resulting aggregates were also composed equally of red and green bacteria (Figure 4B). Similarly, cell-associated aggregation of PAKΔ*popB*-mCherry was restored by co-infection with PAKΔ*STY*-GFP (Figures 4C and D), confirming that T3SS effectors were not required for this process.

Although PAKΔ*fliC* and PAKΔ*pilA* are deficient in binding and motility, they have a functional T3SS [30]. We assessed whether these mutants could restore cell-associated aggregation when co-infected with PAKΔ*popB*. Upon co-infection of PAKΔ*fliC*-mCherry or PAKΔ*pilA*-mCherry with PAKΔ*popB*-GFP, the deficiency in aggregation was restored (Figures S5A–C). Remarkably, each cell-associated aggregate contained a few T3SS-competent bacteria surrounded by many T3SS-deficient bacteria (Figures S5A and B). Together, our findings indicate that the T3SS can function in *trans* to induce formation of cell-associated aggregates and that even a small number of adherent T3SS+ bacteria are sufficient to restore aggregation.

### Cell-associated aggregation is restored by supernatants from PAK-infected cells

**Figure 5:**
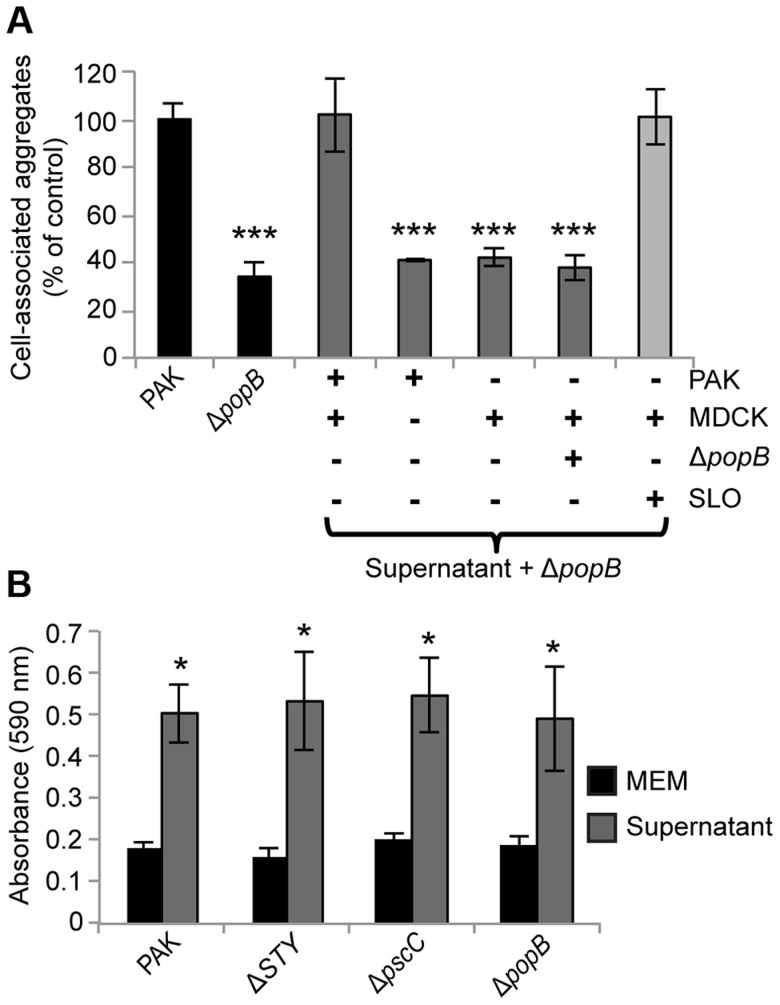
Supernatants from PAK-infected cells restore cell-associated aggregation and enhance abiotic biofilm formation. (A) Cell-associated aggregation by PAKΔ*popB* in the presence of various supernatants was quantified using spinning disk confocal microscopy using the same method as in Figure 2D (n≥3 independent experiments). Data are mean ± SEM. ***p<0.001 compared to PAK. (B) PAK and isogenic T3SS mutants were inoculated into tissue-culture media (MEM) or supernatant from PAK-infected cells and assessed for biofilm formation on microtiter plates (n≥3 independent experiments). Data are mean ± SEM. *p<0.05 compared to control in MEM. Statistics in Supplemental Statistical Analysis (Text S1).

We tested whether supernatants from infected cells were sufficient to restore cell-associated aggregation in PAKΔ*popB*. We co-incubated filtered apical supernatants from PAK-infected MDCK cells with PAKΔ*popB* (Figure S6A) and quantified cell-associated aggregation. Addition of filtered supernatant from PAK-infected cells restored cell-associated aggregation to PAKΔ*popB*, whereas addition of filtered supernatant from bacterial culture only, uninfected cells, or cells infected with PAKΔ*popB*, did not (Figure 5A). These results show that an aggregate-inducing factor is released into the supernatant. Furthermore, the presence of both T3SS+ bacteria and the host cell is required for the generation of this aggregate-inducing factor.

Although the type III translocon has only been shown to insert into eukaryotic cells [31], our results did not distinguish whether the translocon resulted in release of a factor from host cells or from bacteria, nor did they exclude the possibility of a previously undescribed type III-secreted effector. To discriminate among these possibilities, we prepared supernatants from uninfected MDCK cells exposed to the pore-forming toxin streptolysin O (SLO) [32] and tested whether they could restore cell-associated aggregation to PAKΔ*popB* (Figure S6B). Lactate dehydrogenase release assays confirmed that the amount of SLO used was sufficient to permeabilize the apical membrane of the MDCK cells without lysing the cell monolayer (see Materials and Methods). Remarkably, addition of SLO-generated supernatant restored cell-associated aggregation to PAKΔ*popB* (Figure 5A), suggesting that a soluble factor released by host cells in response to pore formation is sufficient to induce aggregate formation.

### The T3SS is not required for abiotic biofilm formation

Our results thus far showed that the T3SS translocon induces release of a host cell factor that leads to biofilm-like aggregate formation on the surface of polarized epithelial cells. We predicted that there should be no effect of the T3SS on biofilm formation in the absence of host cells. To test this hypothesis, we examined the ability of PAK and the isogenic T3SS mutants to form biofilms under two different abiotic conditions: flow chambers and static microtiter plate assays. We found no difference among the mushroom-shaped multicellular biofilm structures formed by PAK, PAKΔ*STY*, PAKΔ*pscC*, and PAKΔ*popB* after 96 hours of growth in flow chambers (Figure S7). We further validated these results in biofilms grown in tissue culture media on microtiter plates and observed no significant difference among PAK, PAKΔ*STY*, PAKΔ*pscC*, and PAKΔ*popB* after 17 hours of growth (Figure 5B, black bars). Collectively, these results show that the T3SS is not required for biofilm formation in the absence of host cells.

### The aggregate-inducing factor enhances abiotic biofilm formation and is sensitive to heat treatment

We assessed whether our isolated aggregate-inducing factor was sufficient to augment biofilm formation in the absence of host cells. Filtered apical supernatants from PAK-infected MDCK cells were added to PAK or the T3SS mutants, and biofilm formation on microtiter plates was measured after 17 hours. Biofilm formation in tissue culture media (MEM) served as the control. The supernatant significantly increased biofilm formation in PAK and in the T3SS mutants (Figure 5B, gray bars), demonstrating that the aggregate-inducing factor can enhance biofilm formation even on abiotic surfaces.

We used this assay to biochemically characterize the aggregate-inducing factor. The ability of supernatants from PAK-infected MDCK cells to enhance biofilm formation was diminished by heat treatment at 95°C but not by protease treatment (Figure S8). Iron has previously been established as a biofilm-enhancing factor, and the iron chelator conalbumin has been shown to reduce *P. aeruginosa* biofilm formation on airway epithelial cells [10], [33]. Addition of conalbumin did not reduce the activity of the aggregate-inducing factor (Figure S8). We thus conclude that the aggregate-inducing factor is neither protein nor iron.

## Discussion

Biofilm formation is increasingly recognized as a major problem in human infections [2], [3]. Because biofilms can resist innate immune defenses and antibiotic therapy, prevention and adequate eradication of biofilms present a difficult clinical challenge. Most studies have examined *P. aeruginosa* biofilms formed on abiotic (i.e. glass or plastic) surfaces, which may not recapitulate important features of host-pathogen interactions. In this work, we establish that co-culture of bacteria with polarized epithelial cells serves as a robust model system of biofilm formation that accurately predicts key aspects of *in vivo* infections. We demonstrate a previously unappreciated role for the type III secretion translocon in biofilm-like aggregate formation on polarized epithelial cells and in a murine model of acute pneumonia.

Several lines of evidence indicated that cell-associated aggregates exhibit key characteristics of biofilms. First, these aggregates contained both extracellular DNA and exopolysaccharides, known components of the extracellular matrix of biofilms. Second, the aggregates showed increased resistance to doses of aminoglycoside that readily kill planktonic bacteria. Third, our results suggest that the flagella and Type IV pili—adhesins that are known requirements for biofilm formation [18], [22]—are also required for cell-associated aggregation. Our cell-associated model system provides a robust tool for investigating many other virulence determinants of biofilms and will be used in future experiments to examine the role of exopolysaccharide secretion, cyclic di-GMP production, and quorum-sensing.

Through quantitative analysis of infected mouse lungs, we uncovered a previously undescribed role for the type III translocon in early *in vivo* infection. Translocon-competent bacteria were more efficient at forming aggregates in the distal airways and alveoli of the lung at 3 hours after infection. While numerous studies clearly show that the type III secreted effectors contribute to virulence [31], the role of the translocon is less well-defined. Previous reports have described translocon-dependent pathogenicity *in vitro* [27], [34]–[36] and *in vivo* [37], although the mechanism remains unclear. Our results also indicate a role for the translocon in the formation of aggregates at early timepoints of infection, which may confer advantages to bacteria, including resistance to antibiotics. However, the inner rod protein of the T3SS has been shown to activate the cytosolic innate immune response and thus may also alert the host to infection [38]. Therefore, early formation of T3SS-dependent aggregates has important implications for the balance between host and pathogen.

Although several *in vitro* studies have reported that the T3SS of *P. aeruginosa* and abiotic biofilm formation are reciprocally regulated [6], [24], [25], we determined that the T3SS is essential for the initiation of cell-associated aggregation in conditions that may recapitulate human infections. These results imply that the coordinate regulation of T3SS and biofilm formation may be more complex during formation of cell-associated aggregation. Indeed, some studies have shown that T3SS can be activated in a subset of biofilm-grown bacteria [7]–[9]. Our data suggest a model in which a few type III secretion-competent bacteria adhere to the apical surface of the epithelial barrier and elicit the release of one or more host cell factors (Figure S9). It is currently unclear whether this release is solely a result of apical cell membrane damage or if it requires downstream signaling from the host cell. The released host cell factor is sufficient for inducing the formation of cell-associated aggregation even in bacteria that lack a functional T3SS. Future studies will examine whether T3SS is downregulated at later time points and whether known regulators of the T3SS in abiotic biofilms, including the RetS/GacS signaling pathway [6], also impact cell-associated aggregation.

Remarkably, our results suggest that this host cell factor has a significant enhancing effect even on biofilms grown on abiotic surfaces. Although we did not investigate *P. aeruginosa* infection of other cell lines, we speculate that the same factor may be responsible for the T3SS-dependent phenomena of “pack-swarming” of *P. aeruginosa* around macrophages [34]. Characterization of the aggregate-inducing factor suggests that it is neither iron nor protein, although it was sensitive to heat treatment. The identity of the host factor and its mechanism of action, which are currently being investigated, may offer new therapeutic strategies for both cell-associated and abiotic biofilms as well as *P. aeruginosa* infection of other types of cells.

Our work is consistent with kinetic measurements of aggregate formation [16] in which time-lapse microscopy showed that cell-associated aggregates develop within minutes after the recruitment of a “sentinel” bacterium, followed by 1–2 more bacteria, and then rapid accumulation of multiple bacteria. Thus, in contrast to abiotic biofilm formation, biofilm-like aggregate formation on the apical surface of polarized epithelial cells is extremely rapid. Our work adds to a growing body of evidence that biofilms may form during the time course of an acute infection. In an analogous *in vitro* model system of *P. aeruginosa* biofilm formation on airway epithelial cells, significant biofilm formation occurred within 3 hours of inoculation [10], while alginate-secreting biofilms have been observed within 8 hours in a mouse model of acute burn infection [39]. Uropathogenic *E. coli* also forms well-organized biofilms in mouse bladders within 6–8 hours [40].

The formation of biofilms during acute infection may have significant implications for medical treatment, as our work suggests that antibiotic resistance develops at early time points of infection. Our results indicate that antibiotic resistance may be mediated by aggregate formation rather than bacterial adaptations—as single bacteria adherent to MDCK cells were readily killed by antibiotics—and are consistent with recently published work that shows that *P. aeruginosa* aggregates formed in stationary-phase culture [41] and in agar gels [42] exhibit increased resistance to antibiotics. Our work further suggests that exopolysaccharide production plays a crucial role in antibiotic resistance, corroborating similar findings in abiotic biofilms [43]. An exopolysaccharide matrix may function in biofilms by binding or sequestering antibiotics, thus limiting penetration into the center of the aggregate.

Previous studies reported that the T3SS is downregulated in biofilms, both in *in vitro* abiotic systems and in longitudinal studies of sputum from cystic fibrosis patients [44]–[46]. These studies have contributed to the prevailing idea that virulence factors required by *P. aeruginosa* for acute infection are incompatible with the ability of *P. aeruginosa* to persist for years within the human host as an antibiotic-resistant biofilm, suggesting that biofilms are only compatible with chronic infections [5], [47]. However, our results demonstrate that T3SS-dependent biofilm formation and antibiotic resistance can develop during early time points of infection. Further delineation of the role of biofilms in acute infections may lead to new therapeutic strategies for treating antibiotic-resistant *P. aeruginosa* infections.

## Materials and Methods

### Ethics statement

This study was carried out in strict accordance with the recommendations in the Guide for the Care and Use of Laboratory Animals of the National Institutes of Health. The protocol was approved by the Institutional Animal Care and Use Committee of Northwestern University (protocol # 2011-1509).

### Bacterial strains and plasmids

The bacterial strains and plasmids used in this study are described in Table 1. Bacterial cultures were streaked from frozen cultures onto Luria-Bertani (LB) agar. For infections, cultures of *P. aeruginosa* were grown in liquid LB overnight shaking at 250 rpm at 37°C in 2 ml LB, diluted 1∶100 into fresh minimal essential medium (MEM), and grown for 2–3 hours to exponential phase. Antibiotic concentrations used were carbenicillin 250 µg/ml and amikacin 400 µg/ml.

**Table 1:** Strains and plasmids used in this study.

Strain or plasmid	Genotype and relevant characteristics	Source or reference
*P. aeruginosa*		
PAK	Wild type	[27]
PAKΔ*exoSTY*	In-frame deletion of *exoS*, *exoT*, and *exoY*	[27]
PAKΔ*pscC*	In-frame deletion of *pscC*	[27]
PAKΔ*popB*	In-frame deletion of *popB*	[27]
PAKΔ*fliC*	In-frame deletion of *fliC*	[57]
PAK*pilA*::Tc^r^	Tetracycline resistance cassette inserted into *pilA*	[58]
PAO1	Wild type	C. Manoil
PAO1Δ*pelFpslD*	In-frame deletion of *pelF* and *pslD*	J.J. Harrison
Plasmids		
pMKB1::mCherry	pMF230 derivative replacing *GFPmut2* with *mCherry* RFP gene; Cb^r^	[59]
pNPT-GFP	Confers constitutive expression of GFP; Cb^r^	B. Kazmierczak

Tc^r^, tetracycline; Cb^r^, carbenicillin.

### Infection of polarized epithelial monolayers

MDCK type II cells or MDCK type II cells stably expressing PH-Akt-GFP [48] were cultured in minimum essential medium (MEM) supplemented with 10% fetal bovine serum at 37°C with 5% CO_2_ [49]. For infections, cells were plated at an instant monolayer density (6×10^5^ cells/well) on 12-mm polycarbonate Transwell filters (0.4 µm pore size; Corning Glassworks, Corning, NY) and cultured for 3 days. Media was changed every 24–48 hours. Exponential phase *P. aeruginosa* (multiplicity of infection (MOI) = 100) was added to the apical chamber of the Transwells for 60 minutes at 37°C. For mixed infection experiments, two different strains of *P. aeruginosa*, one expressing GFP and the other expressing mCherry, were combined (each at an MOI = 50) and immediately inoculated on the apical surface of MDCK cells.

### Bacterial adhesion assays

Bacterial adhesion assays (MOI = 100) were performed on 3-day Transwell-grown MDCK cells in triplicate wells as previously described [50]. Results are reported for three independent experiments with at least six replicates per experiment and are normalized to PAK control (100%).

### Immunofluorescence staining

For confocal microscopy, bacteria were removed at 60 min post-infection by washing the cells three times with PBS. Unless otherwise indicated, the samples were fixed with 4% paraformaldehyde at room temperature for 20 min, permeabilized as necessary with 0.2% Triton X-100 at room temperature for 15 min, and stained as previously described [51]. Actin filaments were stained with CF405M conjugated phalloidin (Biotium) or with Alexa Fluor 350 phalloidin (Invitrogen). To stain extracellular DNA, N, N-Dimethyldodecylamine N-oxide (DDAO, Sigma) was diluted in DMSO to a final concentration of 10 µM, and incubated with fixed samples overnight at 4°C. Fixed samples were subsequently treated with RNase A 50 µg/ml at 37°C for 15 minutes before mounting. For staining mannose-containing exopolysaccharides such as alginate, FITC-Concanavalin A (Sigma) was diluted in water to a final concentration of 100 µg/ml and incubated overnight at 4°C with samples fixed with 2% paraformaldehyde. For staining Psl, FITC-conjugated Hippeastrum Hybrid Lectin Amaryllis (FITC-HHA, EY Laboratories) was diluted to 100 µg/ml and incubated overnight at 4°C with samples fixed with 2% paraformaldehyde. Although Psl has not previously been described in PAK, the *psl* locus is present in the PAK genome [Jorth P and Whiteley M (2013) Genbank accession number KK037225.1].

For experiments to evaluate bacterial viability after exposure to amikacin, MDCK cells were infected apically with log-phase bacteria for 60 min. Side-by-side controls consisted of equal numbers of bacteria incubated in MEM on the apical side of empty Transwell filters. Samples were then treated with amikacin (400 ug/ml) for 2 hours. To distinguish live from dead bacteria, samples were first fixed with 4% paraformaldehyde before staining with the BacLight Bacterial Viability Kit (Invitrogen) according to the manufacturer's instructions. Samples were subsequently treated with RNase A 50 µg/ml at 37°C for 15 minutes before mounting.

### Image acquisition by confocal microscopy

For imaging, Transwell filters were excised with a scalpel and mounted on glass slides using Prolong Gold with or without DAPI (Invitrogen). Unless otherwise indicated, images were acquired using a Nikon FN-1 Microscope equipped with a Nikon C1si spectral confocal, a Nikon Plan 100×/1.4 oil objective, and C1si software (Nikon). For samples stained with DDAO, which required a far-red laser, images were acquired on a Nikon Ti-E microscope equipped with a Yokagawa CSU22 spinning disk confocal and a Nikon Plan Apo VC 100×/1.4 oil objective using Micro-Manager software [52]. Confocal images of bacterial aggregation within murine lungs were acquired with a Zeiss UV LSM510 confocal microscope using a 100× oil objective.

### Quantitative imaging with confocal microscopy

For experiments in which we quantified the efficiency of cell-associated aggregation, images were acquired on a Nikon Ti-E microscope equipped with a Yokagawa CSU22 spinning disk confocal with a Nikon Plan Fluor 40×/1.30 oil objective. Using Micro-Manager software to pre-program a region of ∼5*10^6^ square microns (342 fields) in the center of each specimen, we quantified the number of cell-associated aggregates (defined as groupings of ≥10 bacteria) and normalized them to results from same-day infection with PAK control. Results are reported for three or more independent experiments.

### Confocal image analysis

Orthogonal images were processed and analyzed with NIS Elements software (Nikon). Three-dimensional reconstructions were processed and analyzed with IMARIS software (Bitplane). For co-infection experiments, the relative volume of each strain of bacteria within a single aggregate was determined using ImageJ [53] (NIH) with the 3D Object Counter plug-in [54]. The number of voxels in each channel (red or green) was measured and then calculated as a percentage of total voxels. Results are reported for three independent experiments.

### Infection, fixation, and staining of mouse lungs

Mouse studies of acute pneumonia were conducted using the mouse model described by Comolli *et al* [55]. Six- to eight-week old female BALB/c mice were anesthetized by intraperitoneal injection of a mixture of ketamine (75 mg/kg) and xylazine (5 mg/kg). Mice were intranasally inoculated with 4×10^7^–7×10^7^ CFUs of PAK, PAKΔ*popB*, and PAKΔ*STY* in PBS. Inocula were confirmed by plating serial dilutions on LB agar. For quantification of bacterial persistence at 3 hours post-infection, lungs were excised and homogenized in 5 ml PBS. The bacterial load per lung was determined by plating serial dilutions on Vogel-Bonner-minimal (VBM) agar and incubating at 37°C overnight.

At 3 hour post-infection, the mice were anesthetized and sacrificed by cervical dislocation. Lungs were excised and inflated with approximately 800 ul of 4% paraformaldehyde (PBS, pH 7.4) and placed in a vial of 10 ml 4% paraformaldehyde overnight at room temperature for fixation. Lungs were then placed in a sucrose gradient for cryoprotection prior to sectioning (15% sucrose for 8 h, 30% sucrose overnight at room temperature). Lungs were frozen using Clear Frozen Section Compound (VWR) in a dry ice/isopentane bath. Six-micron sections were cut by the Mouse Histology and Phenotyping Core of the Robert H. Lurie Comprehensive Cancer Center at Northwestern University, supported by the Northwestern University Mouse Histology and Phenotyping Laboratory and a Cancer Center Support Grant (NCI CA060553).

Slides were stored at −80°C prior to additional staining. For visualization of bacterial aggregates, slides were acclimated to room temperature and blocked with 1X TBS, 10% mouse serum, 1% BSA for 2 hours. Rabbit polyclonal anti-Pseudomonas antibody (Abcam ab68538) was diluted 1∶100 and applied for 2 hours at 37°C. Slides were washed with 1X TBS, 0.05% Triton twice for 5 min each. Fluorescently-conjugated Alexa Fluor 555 secondary antibody (Invitrogen) was diluted 1∶1000 and incubated for 1 hour at 37°C. Slides were washed with 1X TBS, 0.05% Triton twice for 5 min each, air-dried, and mounted using Antifade mounting media (Invitrogen).

### Imaging and quantitative analysis of infected murine lungs

For each infected mouse, 5 lung sections from the base of the lungs were imaged at Northwestern University Cell Imaging Facility using a widefield TissueFAXS imaging system (TissueGnostics) with a 40× oil objective. 1400×1000 micron regions from each lung section were randomly selected for imaging. Between 2–10 regions per lung section were imaged, depending on sample quality, for a minimum of 15 images per mouse and at least 60 images per bacterial strain.

For each image, the number of bacteria within aggregates was calculated with the Object Count tool in NIS Elements (Nikon). Image thresholds were set so that a single rod-shaped bacterium measuring 3 µm×1 µm generated a measured area of 2.8 µm^2^ (area = πr^2^+2rh, where r = 0.5 µm and h = 2 µm). Aggregates were defined as objects that contained ≥10 bacteria (or measured ≥28 µm^2^). For each image, percent aggregation was defined as the ratio of bacteria within aggregates to total bacteria.

In calculating bacterial density for each image, we noticed that increased amount of lung tissue resulted in greater numbers of bacteria, presumably due to increased availability of epithelial surfaces upon which bacteria could bind. In contrast, images that contained large empty airspaces and less lung tissue had fewer bacteria. Therefore, bacterial density was defined as the ratio of total bacteria to the amount of lung tissue in each image.

### Cell permeabilization with streptolysin O (SLO)

SLO (Sigma) was reconstituted in PBS and immediately aliquoted and frozen at −80°C to preserve activity. Aliquots were thawed, activated with 10 mM DTT for 30 minutes at 37°C, then immediately placed on ice at 4°C and used within one hour of activation. Three-day Transwell-grown MDCK cells were washed 3 times with cold PBS. SLO (140 µl) was added to the apical surface, and the basolateral medium was replaced with 1.5 ml of cold PBS. Cells were placed on ice and rocked for 10 minutes, then washed twice with ice cold PBS. Room temperature PBS (600 µl for the apical chamber and 1.5 ml for the basolateral chamber of the Transwells) was added and the cells were then incubated at room temperature for 45 minutes to enable pore formation. Supernatants were used immediately. SLO activity was quantified using the Cytotox 96 Assay (Promega), which measures LDH release, according to the manufacturer's instructions. Because SLO activity varies among preparations, we used the amount of SLO required to generate 60–80% cytotoxicity without lysis of the cell monolayer (as determined by measuring basolateral LDH release). For most preparations, this corresponded to approximately 15 µg/ml of SLO. Results are reported for three independent experiments.

### Filtered supernatant experiments

Supernatant (500 µl) from Transwell-grown MDCK cells infected with PAK for 60 minutes was collected by aspiration; residual bacteria was removed by filtration through a 0.22 micron filter (Millipore) and used immediately. Supernatant sterility was confirmed by plating ∼50 µl on LB agar plates. Exponential phase PAKΔ*popB* were pelleted by centrifugation (14000 RPM×5 min), re-suspended in 500 µl of filtered supernatant, and inoculated on the apical surface of Transwell-grown MDCK cells (MOI = 100). Cell-associated aggregation was quantified by spinning disk confocal microscopy. Results are normalized to PAK control (100%) and reported for three or more independent experiments.

To further characterize the aggregate-inducing factor, filtered supernatants were subjected to one of several treatments prior to addition of PAK. Heat treatment used 95°C for 30 minutes. Proteinase K (Sigma) was added at a final concentration of 200 µg/ml to supernatants and incubated for 60 minutes at 37°C. A stock solution of conalbumin (Sigma) was prepared in MEM immediately prior to use and added at a final concentration of 20 µg/ml to supernatants. Biofilm formation was normalized to PAK control with untreated filtered supernatant (100%). Results are reported for three or more independent experiments.

### Flow-chamber biofilm formation

Flow-chamber biofilms were grown in FAB minimal media containing 0.6% glucose. Flow chambers had individual channel dimensions of 1×4×40 mm. The flow system was inoculated by injecting 400 µl of an overnight culture diluted 1∶1000 using a sterile syringe (0.5 ml). Cells were allowed to attach for 1 hour before flow was resumed at 1.75 RPM using a Watson Marlow 205S peristaltic pump. Biofilms were grown for 96 hours before imaging and biomass was estimated. Images were obtained using a laser scanning confocal microscope (Zeiss) using a 63×/1.4 oil objective. 3-D reconstructions were generated using the imaging software program IMARIS (Bitplane). The relative biofilm biomass was measured using the statistical imaging software COMSTAT2. Results are reported for six independent experiments with six images measured per experiment.

### Microtiter plate biofilm formation

*P. aeruginosa* was inoculated into tissue-culture media (MEM) or filtered supernatant from PAK-infected cells and incubated at 37°C for 17 hours. Biofilm formation on microtiter plates was measured as previously described [56]. Results are reported for three independent experiments with at least four replicates per experiment.

### Statistical methods

Statistical significance was calculated using an unpaired two-tailed *t*-test or ANOVA with post-hoc testing when appropriate. Differences were considered to be significant at p values <0.05.

## Supporting Information

Figure S1 **Cell-associated aggregates of PAO1Δ** ***pelFpslD*** **lack exopolysaccharides and show susceptibility to antibiotics.** (A) MDCK cells were infected with PAK-mCherry (red), fixed, and stained for actin (blue) and with FITC-Concanavalin A (green), which binds to mannose-containing polysaccharides. A small amount of FITC-Concanavalin A was bound to MDCK cells as previously reported [12], but at a much lower intensity than in cell-associated aggregates. (B) MDCK cells were infected with PAO1-mCherry (red, left panels) and the exopolysaccharide mutant PAO1Δ*pelFpslD*-mCherry (red, right panels), fixed, and stained for actin (blue). Samples were stained with FITC-HHA (green, top panels), which binds to Psl, and with FITC-Concanavalin A (green, bottom panels). (C) MDCK cells were infected with PAO1Δ*pelFpslD* for 60 minutes and then treated with amikacin (400 ug/ml) for 2 hours. SYTO 9 stained live bacteria (green) and propidium iodide counterstained dead bacteria (red). Representative confocal images are shown. Scale bars, 10 µm.
(TIF)

Figure S2 **PAKΔ** ***fliC*** **and PAKΔ** ***pilA*** **are deficient in cell-associated aggregation.** (A, B) The adhesin mutants PAKΔ*fliC*-mCherry (A) or PAKΔ*pilA*-mCherry (red) (B) bound to the apical surface of MDCK PH-Akt-GFP cells (green) individually or as groups of 2 to 3 bacteria. Representative confocal images are shown. Scale bars, 10 µm. (C) Cell-associated aggregation by PAK and the adhesin mutants PAKΔ*fliC* and PAKΔ*pilA* were quantified using spinning disk confocal microscopy. Shown is the number of aggregates (≥10 bacteria) normalized to PAK (n≥3 independent experiments). Data are mean ± SEM. ***p<0.001 compared to PAK. Statistics in Supplemental Statistical Analysis (Text S1).
(TIF)

Figure S3 (**A**) **PAKΔ** ***pscC*** **is deficient in cell-associated aggregation.** MDCK cells stably transfected with PH-Akt-GFP (green) were infected with PAKΔ*pscC*-mCherry (red, lacks the needle apparatus and translocon) for 60 min, fixed, and stained for actin (blue). (**B**) **T3SS mutants are not deficient in adhesion.** *P. aeruginosa* was added to the apical surface of MDCK cells for one hour. Following washing, bound bacteria were released by detergent and CFUs were enumerated (n≥3 independent experiments). Data are mean ± SEM. There was no statistically significant difference among the strains (p≥0.05), as determined by one-way ANOVA. (**C–F**) **Cell-associated aggregates of PAKΔ** ***popB*** **contain extracellular matrix and are resistant to antibiotics.** MDCK cells were infected with PAKΔ*popB* and stained for (C) extracellular DNA with DDAO (purple), (D) Psl with FITC-HHA (green), and (E) mannose-containing polysaccharide with FITC-ConA (green). (F) After bacterial infection for 60 minutes, samples were treated with amikacin (400 ug/ml) for 2 hours and stained with SYTO 9 for live bacteria (green) and propidium iodide for dead bacteria (red). MDCK cells were stained for actin (blue). Representative confocal images are shown. Scale bars, 10 µm.
(TIF)

Figure S4 **Bacterial aggregation in murine pneumonia requires the T3SS translocon.** BALB/c mice were infected intranasally with PAK, PAKΔ*STY*, or PAKΔ*popB* and lungs were isolated, sectioned, and stained at 3 hours post-infection (n = 3). (**A**) Inoculum at 0 hours of infection (colored circles) and CFUs/lung at 3 hours post-infection are shown for PAK (red squares), PAKΔ*STY* (blue diamonds), and PAKΔ*popB* (green triangles). (**B**) The output/input ratio (CFU/lung to inoculum) was similar (1.1–1.2) for all strains, showing that PAKΔ*popB* was not deficient in growth compared to PAK or PAKΔ*STY*. Data are mean ± SEM. (**C**) Confocal micrographs showed PAK (red) bound to lung epithelium (green), and aggregates could be seen to be composed of numerous bacteria. Representative image from 10 confocal images is shown. Scale bar, 10 µm. (**D**) Widefield epifluorescent images showed more aggregate formation with PAKΔ*STY* than with PAKΔ*popB* (red). Representative images from ≥60 images for each strain are shown. Scale bars, 10 µm. (**E**) The amount of aggregation by PAK and T3SS mutants was quantified and plotted against bacterial density (n≥60 images for each strain). Linear regression lines were applied to each bacterial strain. A composite version of this data is shown in Fig. 3B. The individual graphs are included here for clarity.
(TIF)

Figure S5 **Co-infection with T3SS+ adhesin mutants restores cell-associated aggregation in PAKΔ** ***popB*** **.** MDCK cells were co-infected with equal amounts of mCherry-expressing (red) and GFP-expressing bacteria (green), fixed, and stained for actin (blue). Co-infection with PAKΔ*popB*-mCherry (red) and (A) PAK*ΔfliC*-GFP or (B) PAK*ΔpilA*-GFP (green) resulted in cell-associated aggregates that were composed mostly of PAKΔ*popB*. The relative contribution of each strain to the total cell-associated aggregate was analyzed using volumetric software and depicted as a pie graph (n≥3 independent experiments and ≥5 aggregates per experiment). Data are mean ± SD. (C) Cell-associated aggregation after co-infection was quantified using spinning disk confocal microscopy. Shown is the number of aggregates (≥10 bacteria) normalized to PAK (n≥3 independent experiments). Data are mean ± SEM. ***p<0.001 compared to PAK. Statistics in Supplemental Statistical Analysis (Text S1).
(TIF)

Figure S6 **Depiction of supernatant experimental methods.** (A) Supernatant from MDCK cells infected with PAK (“Infection #1”) was harvested, filtered, and co-incubated with PAKΔ*popB* (“Infection #2”). (B) Supernatant from uninfected MDCK cells treated with streptolysin O (“SLO treatment”) was harvested and co-incubated with PAKΔ*popB* (“Bacterial infection”).
(TIF)

Figure S7 **Flow-cell biofilm formation does not require the T3SS.** (A) GFP-expressing PAK, PAKΔ*STY* PAKΔ*popB*, PAKΔ*pscC*, or PAKΔ*exoSTY* (green) was incubated in flow-chamber cells and biofilm formation was assessed by confocal microscopy after 96 hours. Representative 3-D reconstructions from 6 independent experiments are shown. Scale bars, 30 um. (B) Biofilm biomass was quantified from 36 confocal images (n = 6 independent experiments and 6 images per experiment) after 96 hours of growth in flow chambers. Data are mean ± SEM. There was no statistically significant difference among the strains (p≥0.05), as determined by one-way ANOVA.
(TIF)

Figure S8 **The aggregate-inducing factor is sensitive to heat treatment but insensitive to protease treatment and iron chelation.** PAK was inoculated into tissue-culture media (MEM) or into filtered supernatant from PAK-infected cells that had been treated with heat (95°C for 30 minutes), proteinase K, or the iron chelator conalbumin. Shown is biofilm formation on microtiter plates, normalized to PAK control with untreated filtered supernatant (n≥3 independent experiments). Data are mean ± SEM. ***p<0.001 compared to untreated filtered supernatant. Statistics in Supplemental Statistical Analysis (Text S1).
(TIF)

Figure S9 **Model for the role of T3SS in the formation of biofilm-like aggregates.** Insertion of the type III translocon causes host cell damage and/or triggers host cell signaling. A host cell factor is subsequently released, which induces the formation of cell-associated aggregates. These cell-associated aggregates are encased in an extracellular matrix and show increased resistance to antibiotics.
(TIF)

Text S1 **Supplemental statistical analysis.**
(DOCX)

## References

[1] Costerton JW, Stewart PS, Greenberg EP (1999) Bacterial biofilms: a common cause of persistent infections. Science 284: 1318–1322. 10334980 10.1126/science.284.5418.1318

[2] Hall-Stoodley L, Stoodley P, Kathju S, Hoiby N, Moser C, et al (2012) Towards diagnostic guidelines for biofilm-associated infections. FEMS Immunol Med Microbiol 65: 127–145. 22469292 10.1111/j.1574-695X.2012.00968.x

[3] Parsek MR, Singh PK (2003) Bacterial biofilms: an emerging link to disease pathogenesis. Annu Rev Microbiol 57: 677–701. 14527295 10.1146/annurev.micro.57.030502.090720

[4] Hall-Stoodley L, Costerton JW, Stoodley P (2004) Bacterial biofilms: from the natural environment to infectious diseases. Nature reviews Microbiology 2: 95–108. 15040259 10.1038/nrmicro821

[5] Furukawa S, Kuchma SL, O'Toole GA (2006) Keeping their options open: acute versus persistent infections. Journal of bacteriology 188: 1211–1217. 16452401 10.1128/JB.188.4.1211-1217.2006 PMC1367219

[6] Goodman AL, Kulasekara B, Rietsch A, Boyd D, Smith RS, et al (2004) A signaling network reciprocally regulates genes associated with acute infection and chronic persistence in Pseudomonas aeruginosa. Dev Cell 7: 745–754. 15525535 10.1016/j.devcel.2004.08.020

[7] Horsman SR, Moore RA, Lewenza S (2012) Calcium chelation by alginate activates the type III secretion system in mucoid Pseudomonas aeruginosa biofilms. PLoS One 7: e46826. 23056471 10.1371/journal.pone.0046826 PMC3466208

[8] Manos J, Arthur J, Rose B, Bell S, Tingpej P, et al (2009) Gene expression characteristics of a cystic fibrosis epidemic strain of Pseudomonas aeruginosa during biofilm and planktonic growth. FEMS Microbiol Lett 292: 107–114. 19222585 10.1111/j.1574-6968.2008.01472.x

[9] Mikkelsen H, Bond NJ, Skindersoe ME, Givskov M, Lilley KS, et al (2009) Biofilms and type III secretion are not mutually exclusive in Pseudomonas aeruginosa. Microbiology 155: 687–698. 19246740 10.1099/mic.0.025551-0

[10] Moreau-Marquis S, Bomberger JM, Anderson GG, Swiatecka-Urban A, Ye S, et al (2008) The {Delta}F508-CFTR Mutation Results in Increased Biofilm Formation by P. aeruginosa by Increasing Iron Availability. Am J Physiol Lung Cell Mol Physiol 295: L25–37. 18359885 10.1152/ajplung.00391.2007 PMC2494796

[11] Woodworth BA, Tamashiro E, Bhargave G, Cohen NA, Palmer JN (2008) An in vitro model of Pseudomonas aeruginosa biofilms on viable airway epithelial cell monolayers. Am J Rhinol 22: 235–238. 18588754 10.2500/ajr.2008.22.3178

[12] Bucior I, Mostov K, Engel JN (2010) *Pseudomonas aeruginosa*-mediated damage requires distinct receptors at the apical and basolateral surfaces of the polarized epithelium. Infect Immun 78: 939–953. 20008530 10.1128/IAI.01215-09 PMC2825949

[13] Bucior I, Pielage JF, Engel JN (2012) *Pseudomonas aeruginosa* pili and flagella mediate distinct binding and signaling events at the apical and basolateral surface of airway epithelium. PLoS pathogens 8: e1002616. 22496644 10.1371/journal.ppat.1002616 PMC3320588

[14] Mostov KE (1995) Regulation of protein traffic in polarized epithelial cells. Histol Histopathol 10: 423–431. 7599439

[15] Kierbel A, Gassama-Diagne A, Rocha C, Radoshevich L, Olson J, et al (2007) Pseudomonas aeruginosa exploits a PIP3-dependent pathway to transform apical into basolateral membrane. J Cell Biol 177: 21–27. 17403925 10.1083/jcb.200605142 PMC2064102

[16] Lepanto P, Bryant DM, Rossello J, Datta A, Mostov KE, et al (2011) Pseudomonas aeruginosa interacts with epithelial cells rapidly forming aggregates that are internalized by a Lyn-dependent mechanism. Cellular microbiology 13: 1212–1222. 21615664 10.1111/j.1462-5822.2011.01611.x PMC3813436

[17] Tran C, Eran Y, Ruch T, Bryant D, Datta A, et al (2014) Host cell polarity proteins participate in innate immunity to Pseudomonas aeruginosa infection. Cell Host Microbe in press. 10.1016/j.chom.2014.04.007 PMC4062193 24832456

[18] O'Toole GA, Kolter R (1998) Flagellar and twitching motility are necessary for Pseudomonas aeruginosa biofilm development. Mol Microbiol 30: 295–304. 9791175 10.1046/j.1365-2958.1998.01062.x

[19] Allesen-Holm M, Barken KB, Yang L, Klausen M, Webb JS, et al (2006) A characterization of DNA release in Pseudomonas aeruginosa cultures and biofilms. Mol Microbiol 59: 1114–1128. 16430688 10.1111/j.1365-2958.2005.05008.x

[20] Ma L, Conover M, Lu H, Parsek MR, Bayles K, et al (2009) Assembly and development of the Pseudomonas aeruginosa biofilm matrix. PLoS Pathog 5: e1000354. 19325879 10.1371/journal.ppat.1000354 PMC2654510

[21] Ma L, Lu H, Sprinkle A, Parsek MR, Wozniak DJ (2007) Pseudomonas aeruginosa Psl is a galactose- and mannose-rich exopolysaccharide. J Bacteriol 189: 8353–8356. 17631634 10.1128/JB.00620-07 PMC2168683

[22] Klausen M, Heydorn A, Ragas P, Lambertsen L, Aaes-Jorgensen A, et al (2003) Biofilm formation by Pseudomonas aeruginosa wild type, flagella and type IV pili mutants. Mol Microbiol 48: 1511–1524. 12791135 10.1046/j.1365-2958.2003.03525.x

[23] Engel J, Balachandran P (2009) Role of Pseudomonas aeruginosa type III effectors in disease. Curr Opin Microbiol 12: 61–66. 19168385 10.1016/j.mib.2008.12.007

[24] Kuchma SL, Connolly JP, O'Toole GA (2005) A three-component regulatory system regulates biofilm maturation and type III secretion in Pseudomonas aeruginosa. J Bacteriol 187: 1441–1454. 15687209 10.1128/JB.187.4.1441-1454.2005 PMC545632

[25] Ventre I, Goodman AL, Vallet-Gely I, Vasseur P, Soscia C, et al (2006) Multiple sensors control reciprocal expression of Pseudomonas aeruginosa regulatory RNA and virulence genes. Proc Natl Acad Sci U S A 103: 171–176. 16373506 10.1073/pnas.0507407103 PMC1324988

[26] Watton SJ, Downward J (1999) Akt/PKB localisation and 3′ phosphoinositide generation at sites of epithelial cell-matrix and cell-cell interaction. Curr Biol 9: 433–436. 10226029 10.1016/s0960-9822(99)80192-4

[27] Lee VT, Smith RS, Tummler B, Lory S (2005) Activities of *Pseudomonas aeruginosa* effectors secreted by the Type III secretion system in vitro and during infection. Infect Immun 73: 1695–1705. 15731070 10.1128/IAI.73.3.1695-1705.2005 PMC1064929

[28] Kang PJ, Hauser AR, Apodaca G, Fleiszig SM, Wiener-Kronish J, et al (1997) Identification of Pseudomonas aeruginosa genes required for epithelial cell injury. Mol Microbiol 24: 1249–1262. 9218773 10.1046/j.1365-2958.1997.4311793.x

[29] Diaz MH, Hauser AR (2010) *Pseudomonas aeruginosa* cytotoxin ExoU is injected into phagocytic cells during acute pneumonia. Infect Immun 78: 1447–1456. 20100855 10.1128/IAI.01134-09 PMC2849415

[30] Sundin C, Wolfgang MC, Lory S, Forsberg A, Frithz-Lindsten E (2002) Type IV pili are not specifically required for contact dependent translocation of exoenzymes by Pseudomonas aeruginosa. Microb Pathog 33: 265–277. 12495673 10.1006/mpat.2002.0534

[31] Hauser AR (2009) The type III secretion system of Pseudomonas aeruginosa: infection by injection. Nat Rev Microbiol 7: 654–665. 19680249 10.1038/nrmicro2199 PMC2766515

[32] Bhakdi S, Bayley H, Valeva A, Walev I, Walker B, et al (1996) Staphylococcal alpha-toxin, streptolysin-O, and Escherichia coli hemolysin: prototypes of pore-forming bacterial cytolysins. Arch Microbiol 165: 73–79. 8593102 10.1007/s002030050300

[33] Moreau-Marquis S, O'Toole GA, Stanton BA (2009) Tobramycin and FDA-approved iron chelators eliminate Pseudomonas aeruginosa biofilms on cystic fibrosis cells. Am J Respir Cell Mol Biol 41: 305–313. 19168700 10.1165/rcmb.2008-0299OC PMC2742750

[34] Dacheux D, Goure J, Chabert J, Usson Y, Attree I (2001) Pore-forming activity of type III system-secreted proteins leads to oncosis of *Pseudomonas aeruginosa*-infected macrophages. Mol Microbiol 40: 76–85. 11298277 10.1046/j.1365-2958.2001.02368.x

[35] Goure J, Pastor A, Faudry E, Chabert J, Dessen A, et al (2004) The V antigen of Pseudomonas aeruginosa is required for assembly of the functional PopB/PopD translocation pore in host cell membranes. Infect Immun 72: 4741–4750. 15271936 10.1128/IAI.72.8.4741-4750.2004 PMC470589

[36] Vance RE, Rietsch A, Mekalanos JJ (2005) Role of the type III secreted exoenzymes S, T, and Y in systemic spread of Pseudomonas aeruginosa PAO1 in vivo. Infect Immun 73: 1706–1713. 15731071 10.1128/IAI.73.3.1706-1713.2005 PMC1064930

[37] Galle M, Jin S, Bogaert P, Haegman M, Vandenabeele P, et al (2012) The Pseudomonas aeruginosa type III secretion system has an exotoxon S/T/Y independent pathogenic role during acute lung infection. PLoS One 7: 1–8. 10.1371/journal.pone.0041547 PMC3402384 22844497

[38] Lavoie EG, Wangdi T, Kazmierczak BI (2011) Innate immune responses to Pseudomonas aeruginosa infection. Microbes and infection/Institut Pasteur 13: 1133–1145. 10.1016/j.micinf.2011.07.011 PMC3221798 21839853

[39] Schaber JA, Triffo WJ, Suh SJ, Oliver JW, Hastert MC, et al (2007) Pseudomonas aeruginosa forms biofilms in acute infection independent of cell-to-cell signaling. Infection and immunity 75: 3715–3721. 17562773 10.1128/IAI.00586-07 PMC1952004

[40] Justice SS, Hung C, Theriot JA, Fletcher DA, Anderson GG, et al (2004) Differentiation and developmental pathways of uropathogenic Escherichia coli in urinary tract pathogenesis. Proc Natl Acad Sci U S A 101: 1333–1338. 14739341 10.1073/pnas.0308125100 PMC337053

[41] Alhede M, Kragh KN, Qvortrup K, Allesen-Holm M, van Gennip M, et al (2011) Phenotypes of non-attached Pseudomonas aeruginosa aggregates resemble surface attached biofilm. PLoS One 6: e27943. 22132176 10.1371/journal.pone.0027943 PMC3221681

[42] Staudinger BJ, Muller JF, Halldorsson S, Boles B, Angermeyer A, et al (2014) Conditions associated with the cystic fibrosis defect promote chronic Pseudomonas aeruginosa infection. Am J Respir Crit Care Med 189: 812–824. 24467627 10.1164/rccm.201312-2142OC PMC4225830

[43] Colvin KM, Gordon VD, Murakami K, Borlee BR, Wozniak DJ, et al (2011) The pel polysaccharide can serve a structural and protective role in the biofilm matrix of Pseudomonas aeruginosa. PLoS pathogens 7: e1001264. 21298031 10.1371/journal.ppat.1001264 PMC3029257

[44] Smith EE, Buckley DG, Wu Z, Saenphimmachak C, Hoffman LR, et al (2006) Genetic adaptation by Pseudomonas aeruginosa to the airways of cystic fibrosis patients. Proc Natl Acad Sci U S A 103: 8487–8492. 16687478 10.1073/pnas.0602138103 PMC1482519

[45] Jain M, Bar-Meir M, McColley S, Cullina J, Potter E, et al (2008) Evolution of Pseudomonas aeruginosa type III secretion in cystic fibrosis: a paradigm of chronic infection. Transl Res 152: 257–264. 19059160 10.1016/j.trsl.2008.10.003 PMC2628760

[46] Hu H, Harmer C, Anuj S, Wainwright CE, Manos J, et al (2012) Type 3 secretion system effector genotype and secretion phenotype of longitudinally collected Pseudomonas aeruginosa isolates from young children diagnosed with cystic fibrosis following newborn screening. Clin Microbiol Infect 19 10.1111/j.1469-0691.2012.03770.x 22329595

[47] Nguyen D, Singh PK (2006) Evolving stealth: genetic adaptation of Pseudomonas aeruginosa during cystic fibrosis infections. Proc Natl Acad Sci U S A 103: 8305–8306. 16717189 10.1073/pnas.0602526103 PMC1482488

[48] Yu W, O'Brien LE, Wang F, Bourne H, Mostov KE, et al (2003) Hepatocyte growth factor switches orientation of polarity and mode of movement during morphogenesis of multicellular epithelial structures. Mol Biol Cell 14: 748–763. 12589067 10.1091/mbc.E02-06-0350 PMC150005

[49] Kierbel A, Gassama-Diagne A, Mostov K, Engel JN (2005) The phosphoinositol-3-kinase-protein kinase B/Akt pathway is critical for Pseudomonas aeruginosa strain PAK internalization. Mol Biol Cell 16: 2577–2585. 15772151 10.1091/mbc.E04-08-0717 PMC1087259

[50] Kazmierczak BI, Jou TS, Mostov K, Engel JN (2001) Rho GTPase activity modulates Pseudomonas aeruginosa internalization by epithelial cells. Cell Microbiol 3: 85–98. 11207623 10.1046/j.1462-5822.2001.00091.x

[51] Apodaca G, Katz LA, Mostov KE (1994) Receptor-mediated transcytosis of IgA in MDCK cells is via apical recycling endosomes. Journal of Cell Biology 125: 67–86. 8138576 10.1083/jcb.125.1.67 PMC2120019

[52] Edelstein A, Amodaj N, Hoover K, Vale R, Stuurman N (2010) Computer control of microscopes using microManager. Current protocols in molecular biology/edited by Frederick M Ausubel 10.1002/0471142727.mb1420s92 PMC3065365 20890901

[53] Schneider CA, Rasband WS, Eliceiri KW (2012) NIH Image to ImageJ: 25 years of image analysis. Nature methods 9: 671–675. 22930834 10.1038/nmeth.2089 PMC5554542

[54] Bolte S, Cordelieres FP (2006) A guided tour into subcellular colocalization analysis in light microscopy. J Microsc 224: 213–232. 17210054 10.1111/j.1365-2818.2006.01706.x

[55] Comolli JC, Hauser AR, Waite L, Whitchurch CB, Mattick JS, et al (1999) Pseudomonas aeruginosa gene products PilT and PilU are required for cytotoxicity in vitro and virulence in a mouse model of acute pneumonia. Infect Immun 67: 3625–3630. 10377148 10.1128/iai.67.7.3625-3630.1999 PMC116553

[56] O'Toole GA (2011) Microtiter dish biofilm formation assay. J Vis Exp 47 10.3791/2437 PMC3182663 21307833

[57] Dasgupta N, Wolfgang MC, Goodman AL, Arora SK, Jyot J, et al (2003) A four-tiered transcriptional regulatory circuit controls flagellar biogenesis in Pseudomonas aeruginosa. Mol Microbiol 50: 809–824. 14617143 10.1046/j.1365-2958.2003.03740.x

[58] Watson AA, Mattick JS, Alm RA (1996) Functional expression of heterologous type 4 fimbriae in *Pseudomonas aeruginosa* . Gene 175: 143–150. 8917091 10.1016/0378-1119(96)00140-0

[59] Brannon MK, Davis JM, Mathias JR, Hall CJ, Emerson JC, et al (2009) Pseudomonas aeruginosa Type III secretion system interacts with phagocytes to modulate systemic infection of zebrafish embryos. Cellular microbiology 11: 755–768. 19207728 10.1111/j.1462-5822.2009.01288.x PMC2933946

