# Supplementary figures and images for: The Pseudomonas aeruginosa Type III Translocon Is Required for Biofilm Formation at the Epithelial Barrier
Source: PLoS Pathog. 2014 Nov 6;10(11):e1004479. doi: 10.1371/journal.ppat.1004479 (PMC4223071; doi:10.1371/journal.ppat.1004479)

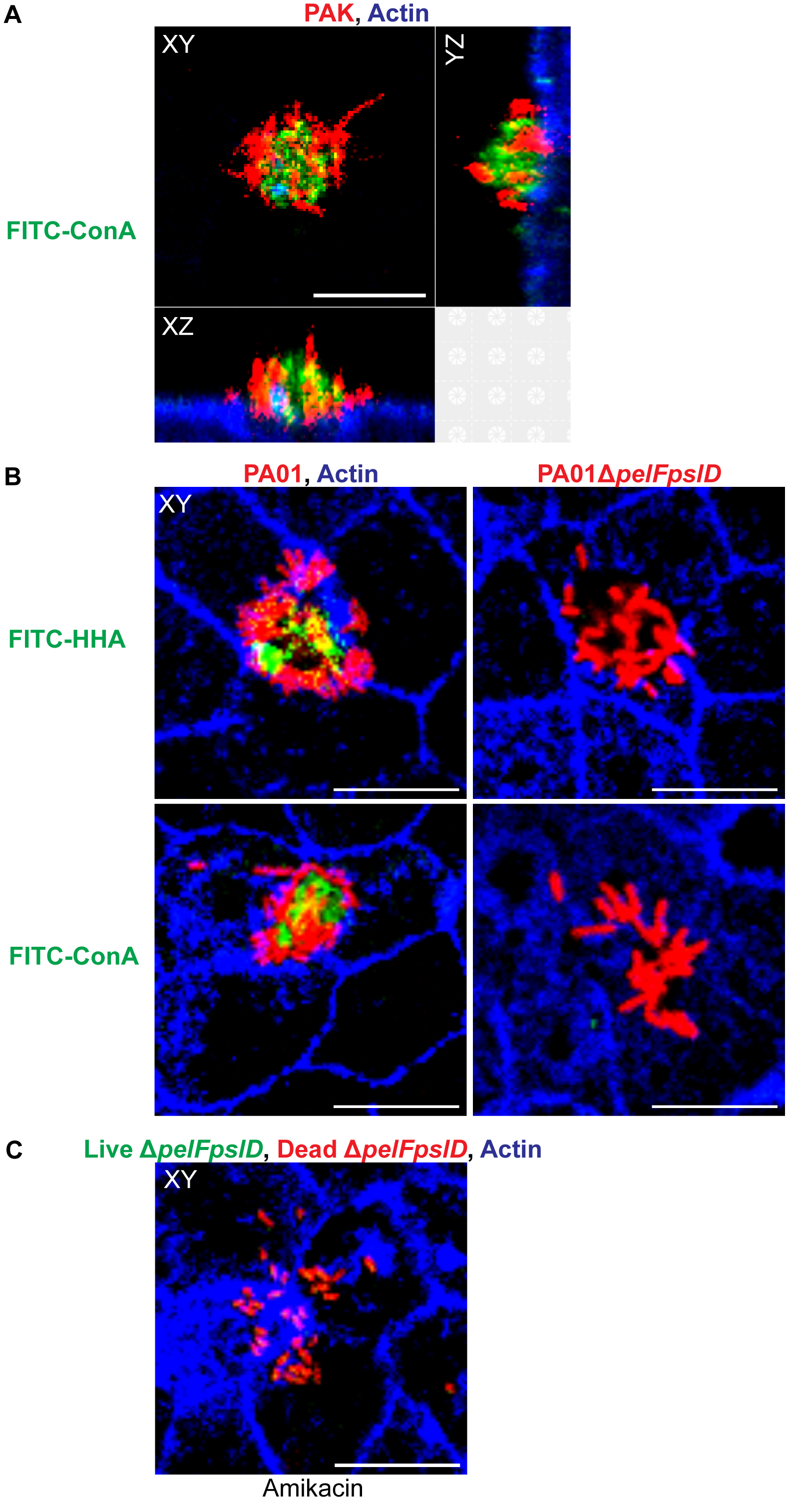

Supplement: Figure S1 — Cell-associated aggregates of PAO1Δ pelFpslD lack exopolysaccharides and show susceptibility to antibiotics. (A) MDCK cells were infected with PAK-mCherry (red), fixed, and stained for actin (blue) and with FITC-Concanavalin A (green), which binds to mannose-containing polysaccharides. A small amount of FITC-Concanavalin A was bound to MDCK cells as previously reported [12], but at a much lower intensity than in cell-associated aggregates. (B) MDCK cells were infected with PAO1-mCherry (red, left panels) and the exopolysaccharide mutant PAO1ΔpelFpslD-mCherry (red, right panels), fixed, and stained for actin (blue). Samples were stained with FITC-HHA (green, top panels), which binds to Psl, and with FITC-Concanavalin A (green, bottom panels). (C) MDCK cells were infected with PAO1ΔpelFpslD for 60 minutes and then treated with amikacin (400 ug/ml) for 2 hours. SYTO 9 stained live bacteria (green) and propidium iodide counterstained dead bacteria (red). Representative confocal images are shown. Scale bars, 10 µm. (TIF) [file ppat.1004479.s001.tif]

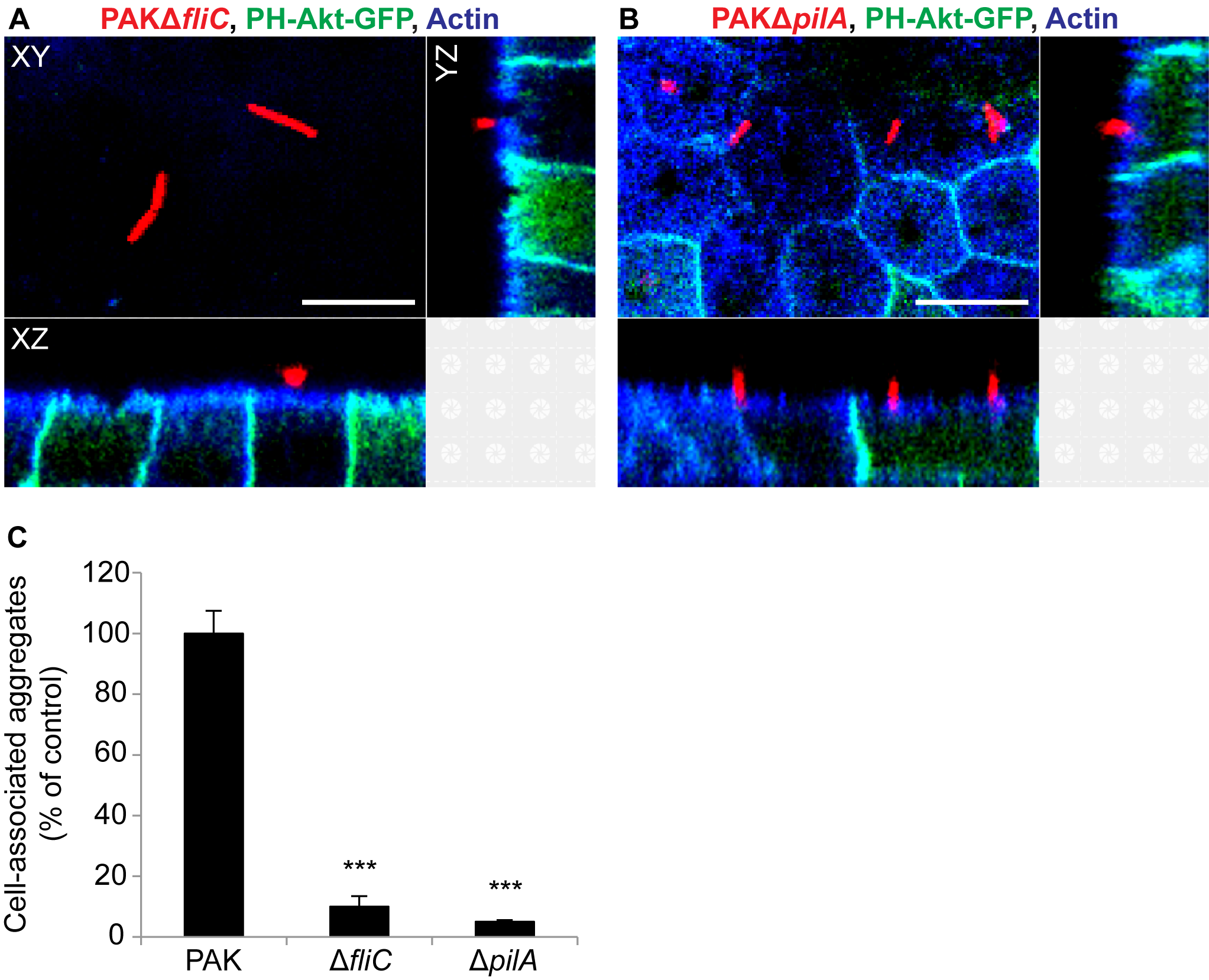

Supplement: Figure S2 — PAKΔ fliC and PAKΔ pilA are deficient in cell-associated aggregation. (A, B) The adhesin mutants PAKΔfliC-mCherry (A) or PAKΔpilA-mCherry (red) (B) bound to the apical surface of MDCK PH-Akt-GFP cells (green) individually or as groups of 2 to 3 bacteria. Representative confocal images are shown. Scale bars, 10 µm. (C) Cell-associated aggregation by PAK and the adhesin mutants PAKΔfliC and PAKΔpilA were quantified using spinning disk confocal microscopy. Shown is the number of aggregates (≥10 bacteria) normalized to PAK (n≥3 independent experiments). Data are mean ± SEM. ***p<0.001 compared to PAK. Statistics in Supplemental Statistical Analysis (Text S1). (TIF) [file ppat.1004479.s002.tif]

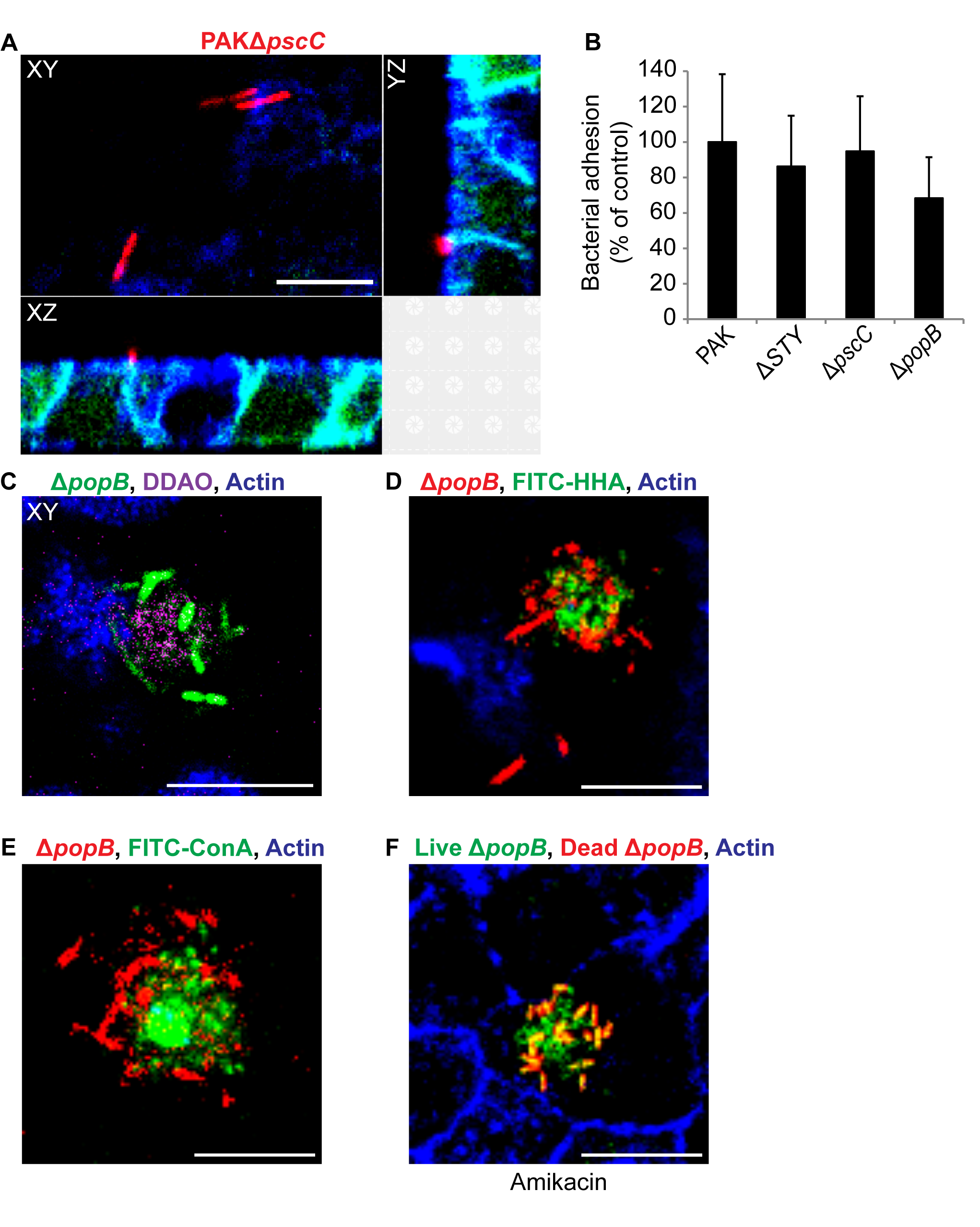

Supplement: Figure S3 — (A) PAKΔ pscC is deficient in cell-associated aggregation. MDCK cells stably transfected with PH-Akt-GFP (green) were infected with PAKΔpscC-mCherry (red, lacks the needle apparatus and translocon) for 60 min, fixed, and stained for actin (blue). (B) T3SS mutants are not deficient in adhesion. P. aeruginosa was added to the apical surface of MDCK cells for one hour. Following washing, bound bacteria were released by detergent and CFUs were enumerated (n≥3 independent experiments). Data are mean ± SEM. There was no statistically significant difference among the strains (p≥0.05), as determined by one-way ANOVA. (C–F) Cell-associated aggregates of PAKΔ popB contain extracellular matrix and are resistant to antibiotics. MDCK cells were infected with PAKΔpopB and stained for (C) extracellular DNA with DDAO (purple), (D) Psl with FITC-HHA (green), and (E) mannose-containing polysaccharide with FITC-ConA (green). (F) After bacterial infection for 60 minutes, samples were treated with amikacin (400 ug/ml) for 2 hours and stained with SYTO 9 for live bacteria (green) and propidium iodide for dead bacteria (red). MDCK cells were stained for actin (blue). Representative confocal images are shown. Scale bars, 10 µm. (TIF) [file ppat.1004479.s003.tif]

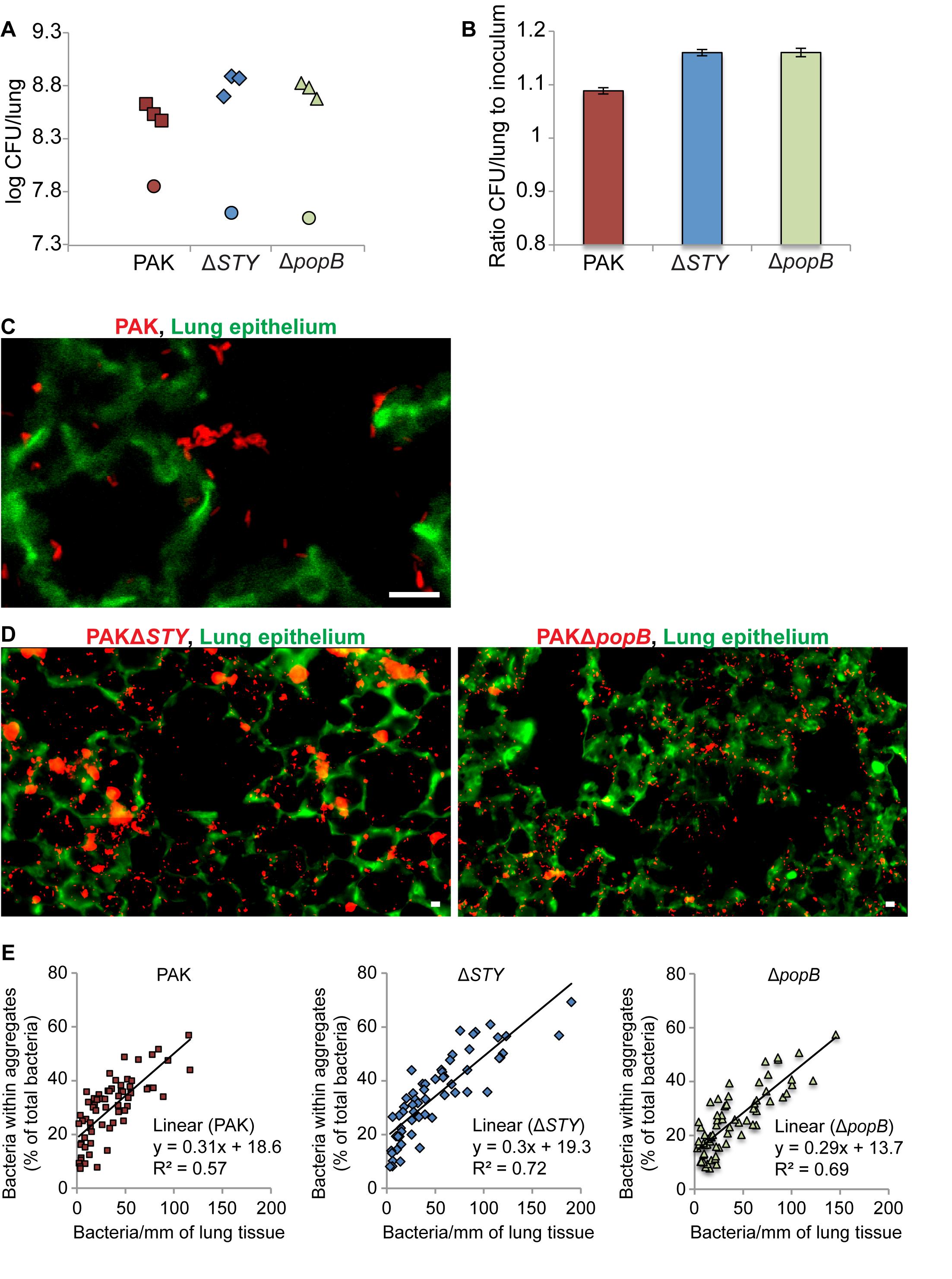

Supplement: Figure S4 — Bacterial aggregation in murine pneumonia requires the T3SS translocon. BALB/c mice were infected intranasally with PAK, PAKΔSTY, or PAKΔpopB and lungs were isolated, sectioned, and stained at 3 hours post-infection (n = 3). (A) Inoculum at 0 hours of infection (colored circles) and CFUs/lung at 3 hours post-infection are shown for PAK (red squares), PAKΔSTY (blue diamonds), and PAKΔpopB (green triangles). (B) The output/input ratio (CFU/lung to inoculum) was similar (1.1–1.2) for all strains, showing that PAKΔpopB was not deficient in growth compared to PAK or PAKΔSTY. Data are mean ± SEM. (C) Confocal micrographs showed PAK (red) bound to lung epithelium (green), and aggregates could be seen to be composed of numerous bacteria. Representative image from 10 confocal images is shown. Scale bar, 10 µm. (D) Widefield epifluorescent images showed more aggregate formation with PAKΔSTY than with PAKΔpopB (red). Representative images from ≥60 images for each strain are shown. Scale bars, 10 µm. (E) The amount of aggregation by PAK and T3SS mutants was quantified and plotted against bacterial density (n≥60 images for each strain). Linear regression lines were applied to each bacterial strain. A composite version of this data is shown in Fig. 3B. The individual graphs are included here for clarity. (TIF) [file ppat.1004479.s004.tif]

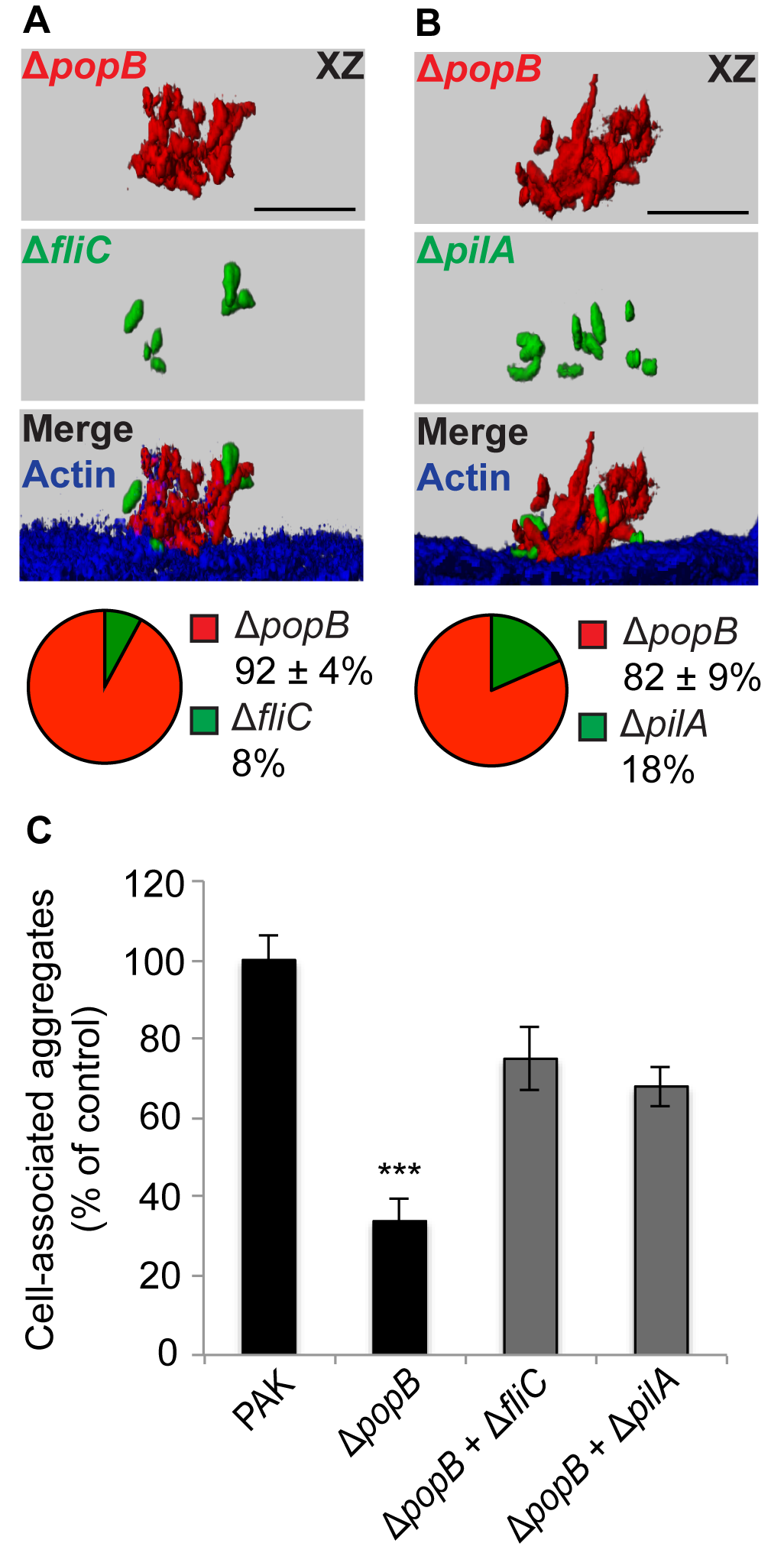

Supplement: Figure S5 — Co-infection with T3SS+ adhesin mutants restores cell-associated aggregation in PAKΔ popB . MDCK cells were co-infected with equal amounts of mCherry-expressing (red) and GFP-expressing bacteria (green), fixed, and stained for actin (blue). Co-infection with PAKΔpopB-mCherry (red) and (A) PAKΔfliC-GFP or (B) PAKΔpilA-GFP (green) resulted in cell-associated aggregates that were composed mostly of PAKΔpopB. The relative contribution of each strain to the total cell-associated aggregate was analyzed using volumetric software and depicted as a pie graph (n≥3 independent experiments and ≥5 aggregates per experiment). Data are mean ± SD. (C) Cell-associated aggregation after co-infection was quantified using spinning disk confocal microscopy. Shown is the number of aggregates (≥10 bacteria) normalized to PAK (n≥3 independent experiments). Data are mean ± SEM. ***p<0.001 compared to PAK. Statistics in Supplemental Statistical Analysis (Text S1). (TIF) [file ppat.1004479.s005.tif]

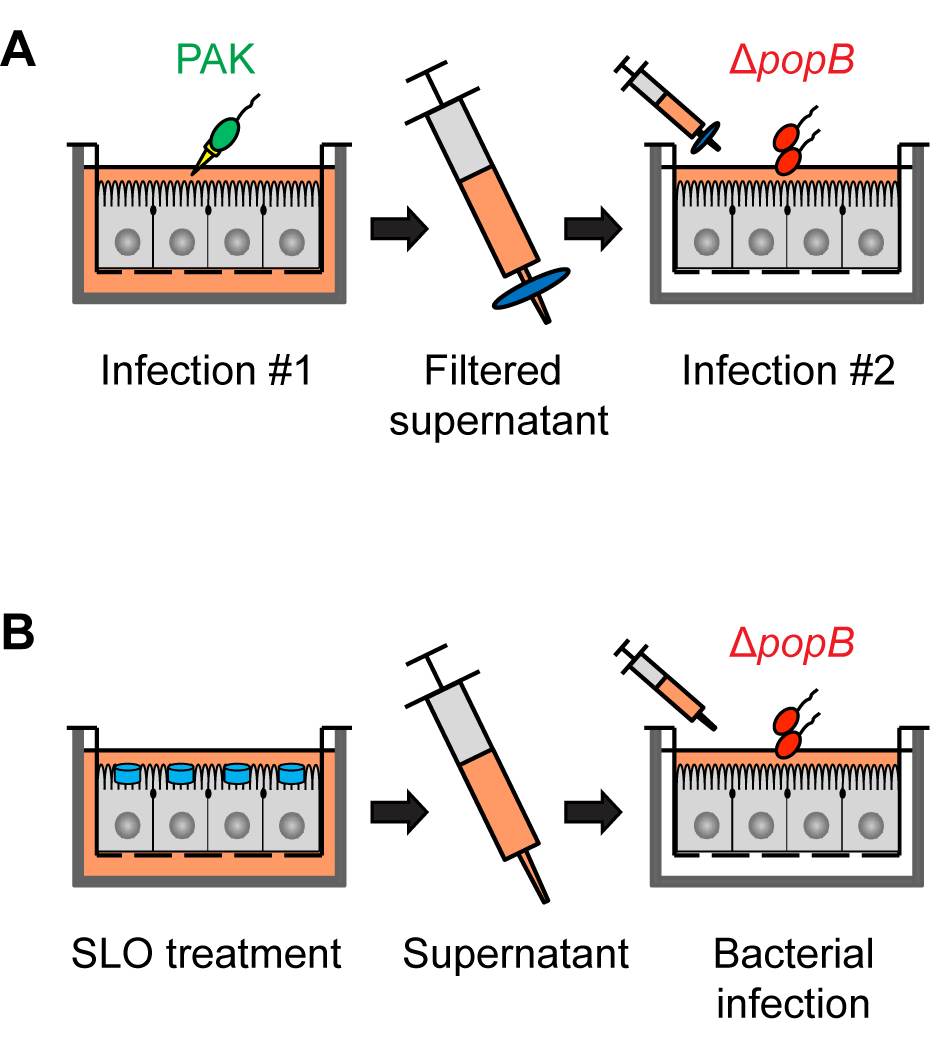

Supplement: Figure S6 — Depiction of supernatant experimental methods. (A) Supernatant from MDCK cells infected with PAK (“Infection #1”) was harvested, filtered, and co-incubated with PAKΔpopB (“Infection #2”). (B) Supernatant from uninfected MDCK cells treated with streptolysin O (“SLO treatment”) was harvested and co-incubated with PAKΔpopB (“Bacterial infection”). (TIF) [file ppat.1004479.s006.tif]

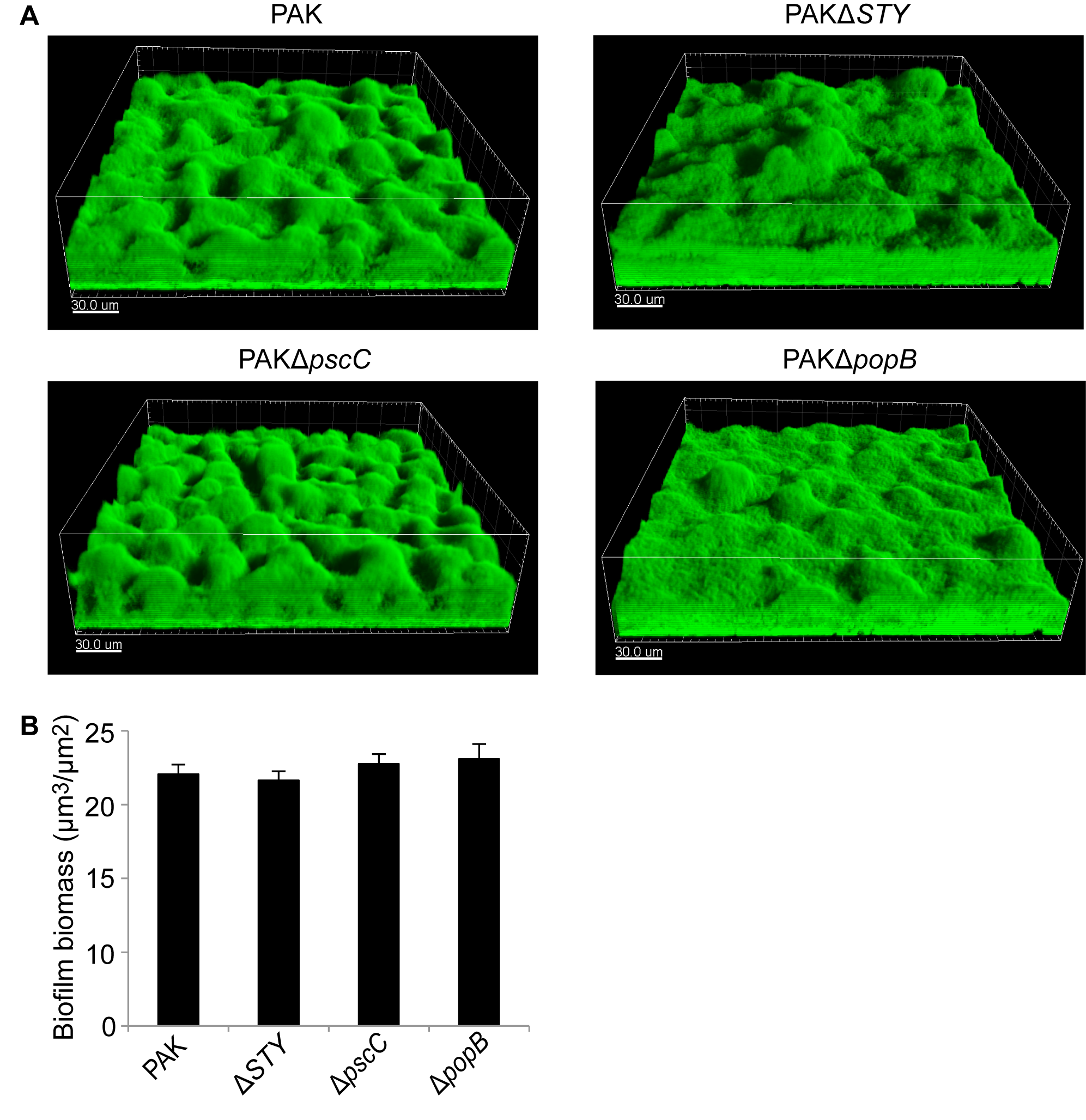

Supplement: Figure S7 — Flow-cell biofilm formation does not require the T3SS. (A) GFP-expressing PAK, PAKΔSTY PAKΔpopB, PAKΔpscC, or PAKΔexoSTY (green) was incubated in flow-chamber cells and biofilm formation was assessed by confocal microscopy after 96 hours. Representative 3-D reconstructions from 6 independent experiments are shown. Scale bars, 30 um. (B) Biofilm biomass was quantified from 36 confocal images (n = 6 independent experiments and 6 images per experiment) after 96 hours of growth in flow chambers. Data are mean ± SEM. There was no statistically significant difference among the strains (p≥0.05), as determined by one-way ANOVA. (TIF) [file ppat.1004479.s007.tif]

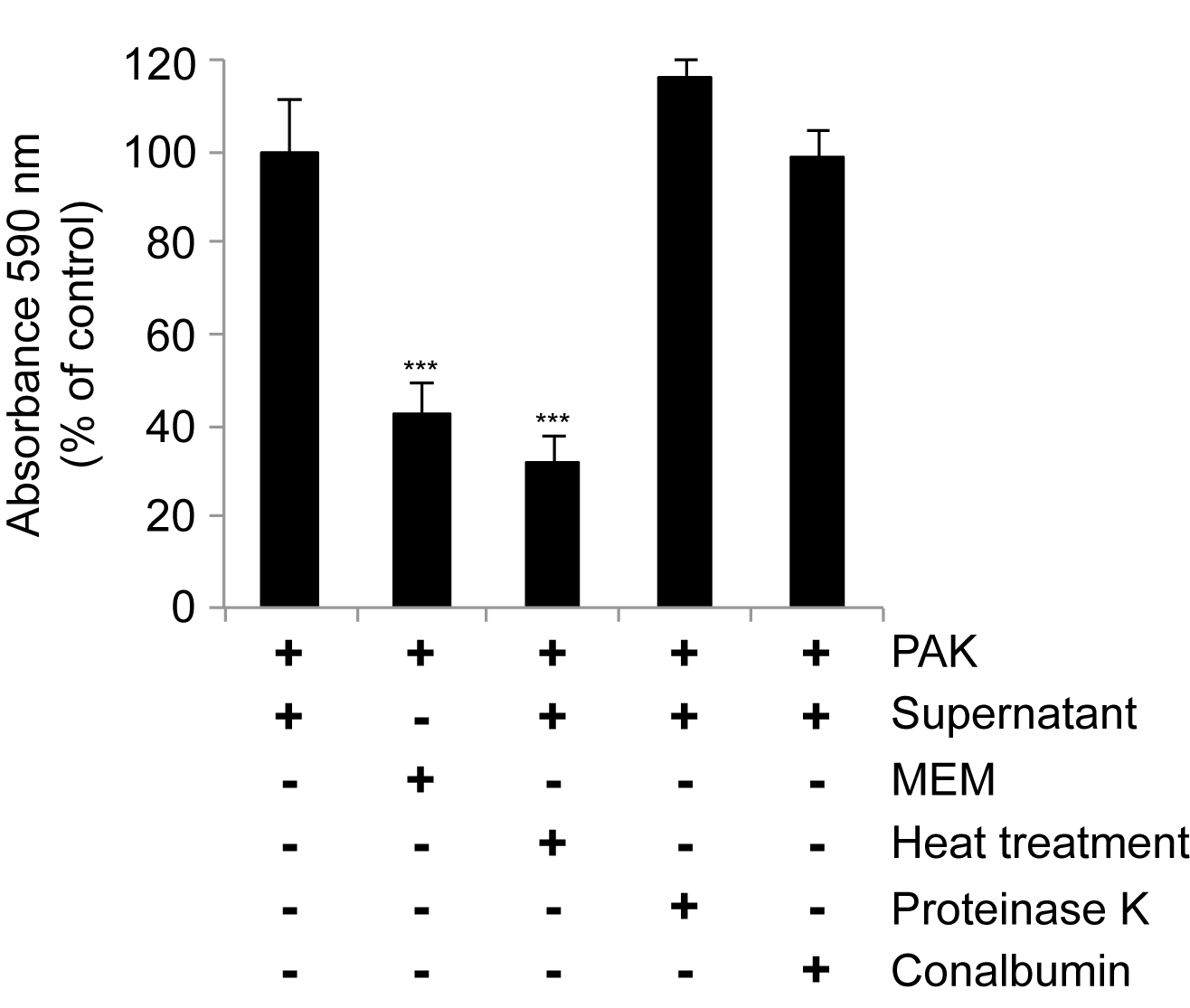

Supplement: Figure S8 — The aggregate-inducing factor is sensitive to heat treatment but insensitive to protease treatment and iron chelation. PAK was inoculated into tissue-culture media (MEM) or into filtered supernatant from PAK-infected cells that had been treated with heat (95°C for 30 minutes), proteinase K, or the iron chelator conalbumin. Shown is biofilm formation on microtiter plates, normalized to PAK control with untreated filtered supernatant (n≥3 independent experiments). Data are mean ± SEM. ***p<0.001 compared to untreated filtered supernatant. Statistics in Supplemental Statistical Analysis (Text S1). (TIF) [file ppat.1004479.s008.tif]

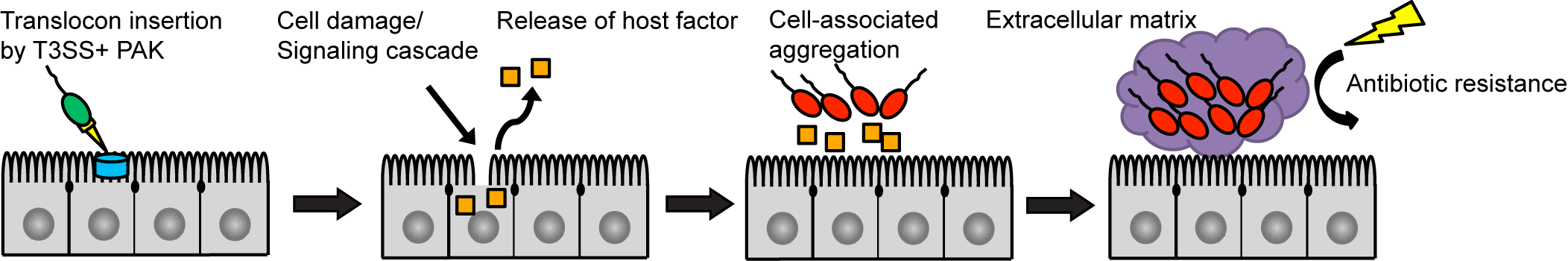

Supplement: Figure S9 — Model for the role of T3SS in the formation of biofilm-like aggregates. Insertion of the type III translocon causes host cell damage and/or triggers host cell signaling. A host cell factor is subsequently released, which induces the formation of cell-associated aggregates. These cell-associated aggregates are encased in an extracellular matrix and show increased resistance to antibiotics. (TIF) [file ppat.1004479.s009.tif]
