# Supplementary material for: The Pseudomonas aeruginosa Type III Translocon Is Required for Biofilm Formation at the Epithelial Barrier
Source: PLoS Pathog. 2014 Nov 6;10(11):e1004479. doi: 10.1371/journal.ppat.1004479 (PMC4223071; doi:10.1371/journal.ppat.1004479)
Supplement: Text S1 — Supplemental statistical analysis. (DOCX) [file ppat.1004479.s010.docx]

**Supplemental Statistical Analysis**

**Fig 2D.** One-way ANOVA showed significant differences among strains for cell-associated aggregation: F(3,22) = 21.86, p < 0.0001.  Two mutants differed significantly from PAK control: p < 0.001, post-hoc Tukey’s test.

**Fig 4D.** One-way ANOVA showed significant differences among conditions for cell-associated aggregation: F(4,26) = 20.29, p < 0.0001.  However, only single infection with Δ*popB* differed significantly from PAK control: p < 0.001, post-hoc Tukey’s test. Co-infections did not differ significantly from PAK control: p > 0.05, post-hoc Tukey’s test.

**Fig 5A.** One-way ANOVA showed significant differences among conditions for cell-associated aggregation: F(6,30) = 17.11, p < 0.0001. Four groups differed significantly from PAK control: p < 0.001, post-hoc Tukey’s test.

**Fig 5B.** Using unpaired two-tailed t-tests, significant differences were detected between:

PAK + MEM and PAK + supernatant: t(4) = 4.52, p = 0.01

PAKΔ*STY* + MEM and PAKΔ*STY* + supernatant: t(4) = 3.08, p = 0.04

PAKΔ*pscC* + MEM and PAKΔ*pscC* + supernatant: t(4) = 3.79, p = 0.02

PAKΔ*popB* + MEM and PAKΔ*popB* + supernatant: t(4) = 2.38, p = 0.04

**Fig S2C.** One-way ANOVA showed significant differences among strains for cell-associated aggregation: F(2,6) = 124.88, p < 0.0001.  Both mutants differed significantly from PAK control: p < 0.001, post-hoc Tukey’s test.

**Fig S3B.** One-way ANOVA showed no difference among strains for overall binding: F(3,8) = 0.21, p = 0.89.

**Fig S5C.** One-way ANOVA showed significant differences among conditions for cell-associated aggregation: F(3,26) = 20.6, p < 0.0001.  However, only single infection with Δ*popB* differed significantly from PAK control: p < 0.001, post-hoc Tukey’s test. Co-infections did not differ significantly from PAK control: p > 0.05, post-hoc Tukey’s test.

**Fig S7B.** One-way ANOVA showed no difference among strains for biofilm formation: F(3,20) = 0.74, p = 0.54.

**Fig S8.** One-way ANOVA showed significant differences among conditions for biofilm formation: F(4,18) = 15.0, p < 0.0001.  However, only biofilm formation with MEM and heat-treated supernatant differed significantly from control with untreated supernatant: p < 0.001, post-hoc Tukey’s test.
